# Non-canonical antigens are the largest fraction of peptides presented by MHC class I in mismatch repair deficient murine colorectal cancer

**DOI:** 10.1186/s13073-023-01275-3

**Published:** 2024-01-19

**Authors:** Giuseppe Rospo, Rosaria Chilà, Vittoria Matafora, Veronica Basso, Simona Lamba, Alice Bartolini, Angela Bachi, Federica Di Nicolantonio, Anna Mondino, Giovanni Germano, Alberto Bardelli

**Affiliations:** 1 Department of Oncology, Molecular Biotechnology Center, University of Torino, Turin, Italy; 2 IFOM ETS - The AIRC Institute of Molecular Oncology, 20139 Milan, Italy; 3 Lymphocyte Activation Unit, Division of Immunology, Transplantation and Infectious Diseases, IRCCS San Raffaele Scientific Institute Via Olgettina, 58, 20132 Milan, Italy; 4 Candiolo Cancer Institute, FPO-IRCCS, 10060 Candiolo, TO Italy; 5 Present address: Boehringer Ingelheim RCV GmbH & Co KG, Vienna, Austria

**Keywords:** Colorectal cancer, Mismatch repair, Neoantigens, Non-coding DNA, Non-canonical antigens, Immune surveillance, Next-generation sequencing, HLA-peptidomics, MAPs

## Abstract

**Background:**

Immunotherapy based on checkpoint inhibitors is highly effective in mismatch repair deficient (MMRd) colorectal cancer (CRC). These tumors carry a high number of mutations, which are predicted to translate into a wide array of neoepitopes; however, a systematic classification of the neoantigen repertoire in MMRd CRC is lacking. Mass spectrometry peptidomics has demonstrated the existence of MHC class I associated peptides (MAPs) originating from non-coding DNA regions. Based on these premises we investigated DNA genomic regions responsible for generating MMRd-induced peptides.

**Methods:**

We exploited mouse CRC models in which the MMR gene *Mlh1* was genetically inactivated. Isogenic cell lines CT26 *Mlh1*^+/+^ and *Mlh1*^-/-^ were inoculated in immunocompromised and immunocompetent mice. Whole genome and RNA sequencing data were generated from samples obtained before and after injection in murine hosts. First, peptide databases were built from transcriptomes of isogenic cell lines. We then compiled a database of peptides lost after tumor cells injection in immunocompetent mice, likely due to immune editing. Liquid chromatography-tandem mass spectrometry (LC-MS/MS) and matched next-generation sequencing databases were employed to identify the DNA regions from which the immune-targeted MAPs originated. Finally, we adopted *in vitro* T cell assays to verify whether MAP-specific T cells were part of the in vivo immune response against *Mlh1*^-/-^ cells.

**Results:**

Whole genome sequencing analyses revealed an unbalanced distribution of immune edited alterations across the genome in *Mlh1*^-/-^ cells grown in immunocompetent mice. Specifically, untranslated (UTR) and coding regions exhibited the largest fraction of mutations leading to highly immunogenic peptides. Moreover, the integrated computational and LC-MS/MS analyses revealed that MAPs originate mainly from atypical translational events in both *Mlh1*^+/+^ and *Mlh1*^-/-^ tumor cells. In addition, mutated MAPs—derived from UTRs and out-of-frame translation of coding regions—were highly enriched in *Mlh1*^*-/-*^ cells. The MAPs trigger T-cell activation in mice primed with *Mlh1*^-/-^ cells.

**Conclusions:**

Our results suggest that—in comparison to MMR proficient CRC—MMRd tumors generate a significantly higher number of non-canonical mutated peptides able to elicit T cell responses. These results reveal the importance of evaluating the diversity of neoepitope repertoire in MMRd tumors.

**Supplementary Information:**

The online version contains supplementary material available at 10.1186/s13073-023-01275-3.

## Background

The mismatch repair (MMR) system is a mechanism able to detect and correct erroneous substitutions, such as single-nucleotide variants (SNVs), insertions and deletions (indels) following DNA replication [1]. About 15% of stage I–III colorectal cancers (CRCs) and 4–5% of metastatic CRC present deregulation of the MMR machinery [2]. Based on the MMR status, CRCs are classified as mismatch repair proficient (MMRp) and mismatch repair deficient (MMRd) [3]. MMRp tumors are the vast majority of CRCs and are referred to as microsatellite stable (MSS) tumors. In contrast, MMRd tumors display a shifting length of microsatellites and are classified as microsatellite unstable (MSI) tumors [4, 5]. The inefficient DNA repair system leads MMRd tumors to accumulate a 10-fold increase of alterations across the genome compared to MMRp tumors [6–8]. Those alterations, if transcribed and translated, can be presented as peptides by the major histocompatibility complex (MHC) class I and II and trigger adaptive immunity [9, 10].

The hypermutation status of MSI tumors is associated with increased responsiveness to immune-based therapies, such as immune checkpoint blockades (ICBs) [11–13]. The Food and Drug Administration (FDA) approved for the first time the tissue agnostic use of pembrolizumab based on MSI molecular status [14].

Although the contribution of neoantigens in deciphering the immunogenic features of these tumors has been well described, the extent to which the non-coding portions of the genome affects the immunogenicity of MMRd tumors is largely unknown. Indeed, a variety of non-coding regions can contribute to the repertoire of tumor antigens, including novel or unannotated open reading frames [15], retained introns [16], long noncoding RNAs [17], untranslated regions (UTRs) [18, 19], junctions, and intergenic regions [20, 21]. Interestingly, MHC class I associated peptides (MAPs) originating from non-coding portions of the genome were shown to be potential immunogenic targets of T lymphocytes [21, 22]. MAPs can also derive from a variety of genetic and epigenetic changes leading to the transcription and translation of genomic sequences normally not expressed in cells or from non-canonical open reading frames that emerge in tumor cells [23, 24].

Given the potential relevance of non-coding MAPs and mutated MAPs (mMAPs) in the immunogenic properties of MMRd tumors, in this work, we systematically analyzed how the immune system could perturb the canonical and non-canonical antigen repertoire of MMRd and MMRp tumors. Considering the challenges in functionally characterizing these aspects in human models, we exploited a well-characterized isogenic murine CRC model in which we previously perturbed MMR proficiency through *Mlh1* gene knock-out with the CRISPR/Cas9 technology [10]. Whole genome sequencing (WGS) and RNA sequencing (RNAseq) data from tumors before and after immune editing by the hosts allowed us to establish a database of peptides that could potentially trigger an immune response. Then, the database was combined with a liquid chromatography pipeline coupled with tandem mass spectrometry (LC-MS/MS), thus allowing identification of the peptides directly eluted from MHC class I complex [25].

WGS analysis of CT26 *Mlh1*^*-/-*^ excised from immunocompromised and immunocompetent mice showed a diverse spread of targeted variants that are mostly accumulated in UTR and coding regions. In addition, MMRd cells displayed a great fraction of targeted mMAPs derived from SNVs and indels in UTR and out-of-frame translation of coding regions, which were the most abundant at the transcript level. Ultimately*, in vitro* peptide-driven expansion, and restimulation, revealed evidence of MAP-specific T cell responses in mice that had rejected MMRd tumors.

Since 99% of cancer mutations are in non-coding regions [26], we postulate that non-coding DNA could be a source of novel MAPs and contribute to the high immunogenicity of MSI tumors when treated with ICB. We provide a proof-of-concept that in MMRd tumors, the non-coding portions of the genome can relevantly contribute to prompting an immune response in cancer patients and may potentially be exploited to predict tumors likely to respond to immune-based therapies.

## Methods

### Cell line

CT26 is a chemically induced colon carcinoma derived from BALB/c mice; CT26 cells were cultured in RPMI 1640, 10% FBS, 1% glutamine, 1% penicillin and streptomycin (Sigma Aldrich). Cells were regularly checked for mycoplasma contamination and before performing the genome editing experiments, they were injected into matched syngeneic mice to ensure cell tumorigenicity. After tumor formation, we established again *in vitro* cell cultures. All cells underwent WGS.

### Gene editing

To knockout the *Mlh1* gene in CT26 cells, we used the genome editing one vector system (lentiCRISPR-v2) (Addgene #52961) as previously reported [10]. Briefly, sgRNAs were designed using the CRISPR tool (http://crispr.mit.edu) to minimize potential off-target effects. For transient expression of CRISPR-Cas9 system, we transfected cells with lentiCRISPR-v2 vector plasmid (same guides as previously described) [10]. Transfection was carried out using Lipofectamine 3000 (Life technologies) and Opti-MEM (Invitrogen), according to the manufacturer’s instructions. After 48 h, cells were incubated with puromycin (Sigma Aldrich) for 2 days and subsequently, single cell dilution was performed in 96-well plates. The absence of Mlh1 and Cas9 was confirmed by western blot [10].

### Animal studies

All animal experiments were carried out according to the protocols (21635.14 and 75DA4.175) approved by the Institutional Animal Care and the Italian Ministry of Health. All the experiments were performed in accordance with international law and policies. Four- to six-week-old female NOD-SCID and BALB/c mice were purchased from Charles River and were maintained in pathogen-free conditions in individually ventilated cages. CT26 cells were resuspended in PBS and injected (5×10^5^ cells per mouse) subcutaneously 150 days after genome editing. When tumors reached the experimental endpoint, mice were euthanized with carbon dioxide and tumors were explanted for subsequent analyses.

### Hybridomas and antibodies

HB-79 (producing anti H-2Kd/H-2Dd mouse IgG2a) and HB-27 (producing anti H-2Ld mouse IgG2a) hybridoma cell lines were purchased from ATCC and grown in Iscove medium (Sigma) supplemented with 10% FBS. Hybridoma were then adapted to protein-free PFHM medium (Thermo) for expansion and conditioning. Once cells were dead, the medium containing immunoglobulins was centrifuged and filtered to be run on a MabSelect Sure (ProteinA) column (Cytiva) mounted on Akta Pure (Cytiva). IgGs were then eluted at acid pH and dialyzed against physiologic storage buffer.

### Immune-peptidomic workflow

Six CT26 *Mlh1*^*+/+*^ and six CT26 *Mlh1*^*-/-*^ tumor masses were explanted from NOD-SCID mice and manually smashed with disposable micro tissue homogenizers in lysis buffer solution (0.25% sodium deoxycholate, 0.2 mM iodoacetamide, 1 mM EDTA, 1:200 protease inhibitors cocktail, 1 mM PMSF, 1% octyl-b-D glucopyranoside in PBS). Proteins were extracted for 1 h at 4°C in continuous mixing, then samples were centrifuged at 30,000 rpm for 1 h at 4°C. Protein extracts in the supernatants were pre-cleared with 1 mL of protein A resin (GenScript) for 1 h at 4°C in agitation, then dosed by BCA assay. Around 20 mg were used for reaction, and each experiment was performed three times.

Protein A resin was washed three times with PBS, then resuspended in PBS-Tween 0.01% and added with 5 mg of anti H-2Kd/H-2Dd or anti H-2Ld antibodies. Control samples without antibody were included. Resin and antibody were left with continuous mixing at 4°C overnight, then the unbound antibody was discarded. Antibodies and resins were crosslinked with 5 mM disuccinimidyl suberate for 1 h at room temperature with continuous mixing, then the reaction was quenched with 1 M Tris HCl pH 7.5 for 1 h at room temperature with continuous mixing.

H-2Ld was immunoprecipitated from precleared proteins by continuous mixing with crosslinked resin/antibody at 4°C overnight, then the unbound protein extract was subsequently passed on the following crosslinked resin/antibody to immunoprecipitate H-2Kd/H-2Dd at 4°C overnight. The resins were washed and centrifuged for two times with 10 volumes of 400 mM NaCl, 20 mM Tris-HCl, 0.2% NP40 then with 15 volumes 20 mM Tris-HCl pH 8, 3 min each wash. Peptides were eluted from H-2 complexes with eight washes in TFA 0.2%, 1 min each. Supernatants were passed through an Amicon Ultra 0.5 mL 3k filter to separate H-2 molecules from the peptides.

Peptides in 0.2% TFA were dried by vacuum centrifugation, solubilized in 5% formic acid, and purified by binding to disposable reversed-phase C18 stage tips. Samples were injected onto a quadrupole Orbitrap Q-exactive HF mass spectrometer (Thermo Scientific), each one in technical duplicate. Peptides separation was achieved on a linear gradient from 95% solvent A (2% acetonitrile, 0.1% formic acid) to 55% solvent B (80% acetonitrile, 0.1% formic acid) over 120 min and from 55 to 100% solvent B in 3 min at a constant flow rate of 0.25 μl/min on UHPLC Easy-nLC 1000 (Thermo Scientific) where the liquid chromatography system was connected to a 23-cm fused-silica emitter of 75-μm inner diameter (New Objective, Inc. Woburn, MA, USA), packed in-house with ReproSil-Pur C18-AQ 1.9-μm beads (Dr Maisch Gmbh, Ammerbuch, Germany) using a high-pressure bomb loader (Proxeon, Odense, Denmark). The mass spectrometer was operated in data-dependent acquisition (DDA) mode as described previously [27]: dynamic exclusion enabled (exclusion duration = 15 seconds), MS1 resolution = 70,000, MS1 automatic gain control target = 3 × 10^6^, MS1 maximum fill time = 60 ms, MS2 resolution = 17,500, MS2 automatic gain control target = 1 × 10^5^, MS^2^ maximum fill time = 60 ms, and MS2 normalized collision energy = 25. For each cycle, one full MS1 scan range = 300–1650 m/z, was followed by 12 MS^2^ scans using an isolation window of 2.0 m/z.

The MS data were analyzed using MaxQuant with 1% false discovery rate (FDR). Peptides were searched against the uniport-proteome_Mouse_010419 database or the customized reference databases that contained the sequences identified by RNAseq data. The identification was performed as follows: peptides eluted from the CT26 *Mlh1*^*+/+*^ sample were matched to both the *Mlh1*^*+/+*^ and *Mlh1*^*-/-*^ custom databases. Peptides eluted from the *Mlh1*^*-/-*^ sample were compared to both the CT26 *Mlh1*^*+/+*^ and *Mlh1*^*-/-*^ custom databases and only peptides identified in the *Mlh1*^*-/-*^ database were selected. N-term acetylation and methionine oxidation were set as variable modifications. Enzyme specificity was set as unspecific when peptides were searched against the UniProt mouse database, while enzyme specificity was set as no enzyme when peptides were searched against customized reference databases and peptides FDR was set to 0.01.

### Whole genome sequencing analysis

Genomic DNA (gDNA) was extracted from BALB/c tissue, *Mlh1*^*+/+*^ and *Mlh1*^*-/-*^ cell lines using ReliaPrep gDNA tissue miniprep system (Promega). Starting from 500 ng of gDNA, next-generation sequencing (NGS) libraries were prepared in house by means of Nextera DNA Flex Library Prep kit (Illumina Inc., San Diego, CA, USA), according to the manufacturer’s protocol. Quality of libraries was checked with High-Sensitivity DNA assay kit (Agilent Technologies, Santa Clara, CA), while DNA fragments’ size distribution was assessed using the 2100 Bioanalyzer with a Qubit dsDNA Quantification Assay Kits (ThermoFisher Scientific, Waltham, MA USA). Equal amounts of final DNA libraries were pooled and sequenced on NovaSeq 6000 (Illumina Inc., San Diego, CA, USA) as paired-end 150 bp reads at IntegraGen SA (Evry, France) and FastQ files were generated using bcl2fastq v2.17 software. Genomic analyses were performed using a bioinformatic pipeline previously described [28]. On average, sequenced samples reached a median depth of 93× (Table 1). CT26 *Mlh1*^*+/+*^ and *Mlh1*^*-/-*^ mutational calling was performed subtracting BALB/c germline variants. Only genomic positions present with a minimum depth of 10× and supported by at least nine mutated reads were examined. To annotate alterations (SNVs and indels) at genomic level, a browser extensible data (BED) file was built that included all genomic regions. Coding, intronic, and UTR regions BED files were downloaded from the University of California Santa Cruz (UCSC) table browser (assembly: mm10; table: refFlat). Non-coding RNA (ncRNA) regions were extrapolated from the whole mm10 refFlat table filtering for *cdsEnd* − *cdsStart* = 0. Each of those specific region BED files was further processed with the *bedtools merge* command [29]. To generate the BED file for the extragenic regions, the previously merged BED files were concatenated and subtracted from the whole mm10 chromosome annotation tracks. The size of each region was calculated using the *bedtools coverage -hist* command. The combination of coding, intronic, and UTR merged tracks together with the extragenic regions BED file was employed for the mutational annotation. Normalized tumor mutational burden (TMB) was evaluated as the number of variants per megabase (Mb) considering those derived from each specific region. The analysis of targeted mutations was performed calculating for each region the natural logarithm of normalized ratios between post- and pre-injection TMB. MSI score was calculated using MSIsensor-pro [30]. Microsatellite indels were calculated by matching the indel calls and mutated loci defined by the MSIsensor pipeline.

**Table 1:** List of WGS analyses performed in CT26 samples. Sequencing features of BALB/c mouse strain, *Mlh1*^*+/+*^ and *Mlh1*^*-/-*^ samples. The following data are described in the table: total number of sequenced reads, number of mapped reads on the reference genome, and whole genome median depth

Sample	Reads	Mapped	Median depth
BALB/c	1078015820	99.56%	105
CT26 *Mlh1*^*+/+*^	958072282	99.61%	91
CT26 *Mlh1*^*+/+*^ post BALB/c M1	877846209	99.66%	83
CT26 *Mlh1*^*+/+*^ post BALB/c M2	856369151	99.63%	80
CT26 *Mlh1*^*+/+*^ post BALB/c M3	1088138369	99.75%	103
CT26 *Mlh1*^*+/+*^post NOD-SCID M1	915507037	99.61%	85
CT26 *Mlh1*^*+/+*^post NOD-SCID M2	964605317	99.62%	92
CT26 *Mlh1*^*+/+*^post NOD-SCID M3	888087399	99.58%	82
CT26 *Mlh1*^*-/-*^	1083850382	99.71%	104
CT26 *Mlh1*^*-/-*^ post BALB/c M2	996468559	99.59%	90
CT26 *Mlh1*^*-/-*^ post BALB/c M6	1038671151	99.52%	94
CT26 *Mlh1*^*-/-*^ post BALB/c M7	1089980019	99.63%	106
CT26 *Mlh1*^*-/-*^ post NOD-SCID M5	1022739553	99.63%	100
CT26 *Mlh1*^*-/-*^ post NOD-SCID M7	854313322	99.59%	81

### RNA sequencing analysis

Total RNA was extracted from CT26 *Mlh1*^*+/+*^ and CT26 *Mlh1*^*-/-*^ cells using Maxwell® RSC miRNA Tissue Kit (AS1460, Promega), according to the manufacturer’s protocol. The quantification of RNA was performed by DeNovix Ds-11 Spectrophotometer (Resnova) and Qubit 3.0 Fluorometer (Life Technologies). RNA integrity was evaluated with Agilent 2100 Bioanalyzer using the Agilent RNA 6000 Nano Kit; 500 ng of total RNA, with RNA integrity number (RIN) score between 8 and 10, was used for NGS Library using TruSeq Stranded mRNA Library Preparation Kit LP (48 samples) according to the manufacturer’s protocol. The standard RNA fragmentation profile was used (94 °C for 8 min). PCR-amplified RNAseq library quality was assessed using the Agilent DNA 1000 kit on the Agilent 2100 BioAnalyzer and quantified using Qubit 3.0 Fluorometer (Life Technologies). Libraries were diluted to 10 nM using Tris-HCl (10 mM pH 8.5) and then pooled together. The 7.5 pM diluted pool was run on MiSeq to evaluate library quality and balancing. Rebalanced pool was denatured according to the NextSeq system guide, and 1.3 pM were run on NextSeq500 using NextSeq 500/550 High Output v2.5 kit (150 cycles).

To calculate the coverage over-depth data, single-end FastQ files were processed as follows: files were aligned with MapSplice v2.2.0 [31] using mm10 assembly as reference genome. The generated alignment files were handled to translate genomic coordinates to transcriptomic ones and to filter out alignments carrying indels using the *sam-xlate* and *sam-filter* commands from UNC-Chapel Hill Bioinformatics Utilities*.* The final compressed sequence alignment/map (BAM) files were inspected through the *bedtools genomecov* command using *-bga* and *-split* as parameters [29]. The generated files were further analyzed using the *bedtools intersect* command [29] to count for every genomic region the number of bases covered for each minimum depth value. For each region, the count of annotated MAPs was normalized using the number of bases covered with at least 10× depth.

To calculate the peptide transcripts per million (TPM), the following formula was applied for each region:

$$\textrm{TPM}=\frac{P}{\sum (P)}\times {10}^6$$

where

$$P=\frac{\textrm{number}\ \textrm{of}\ \textrm{MAPs}\ \textrm{supporting}\ \textrm{reads}\ \textrm{mapped}\ \textrm{to}\ \textrm{each}\ \textrm{region}\times 10^{3}}{\textrm{region}\ \textrm{length}\ \textrm{in}\ \textrm{base}\ \textrm{pair}}.$$

### Generation of specific peptide database for mass spectrometry data search

Each sequence contained in the FastQ files generated during the RNAseq experiment (Table 2) was subjected to a six-frame translation: the three possible reading frames in both directions of the strand. All the translated sequences were divided into KMERs of length 8–11 and then uniquely counted. The *Mlh1*^*+/+*^ specific database was built including KMERs (peptides) that exhibited at least 10 counts at the time of injection and after excision from the RNAseq of immunocompromised mouse (NOD-SCID) and that disappeared in tumor masses obtained from the RNAseq of immunocompetent mouse (BALB/c). The *Mlh1*^*-/-*^ custom database was assembled as follows: first peptides that showed at least 10 counts at the time injection and retained after excision from the RNAseq of immunocompromised mouse were selected; then those peptides were compared to the sequences obtained from the RNAseq of tumors grown in three immunocompetent mice. Peptide lost or strongly counter selected in at least one BALB/c tumor, measured as $\left(\textrm{count}{\textrm{s}}_{\textrm{BALB}/\textrm{c}}=0\ \textrm{or}\ \left(\textrm{count}{\textrm{s}}_{\textrm{pre}-\textrm{selected}}-\textrm{count}{\textrm{s}}_{\textrm{BALB}/\textrm{c}}\ge 10\ \textrm{and}\ \frac{\textrm{count}{\textrm{s}}_{\textrm{pre}-\textrm{selected}}}{\textrm{count}{\textrm{s}}_{\textrm{BALB}/\textrm{c}}}\ge 10\right)\ \right)$, were further selected. Peptide sequences found in *Mlh1*^*+/+*^ tumors excised from BALB/c and NOD-SCID were excluded from the *Mlh1*^*-/-*^ specific database as well as sequences present in *Mlh1*^*+/+*^ cells or strongly expanded in *Mlh1*^*-/-*^ compared to the *Mlh1*^*+/+*^ counterpart. The latter measure was calculated as follows:

$$\left(\textrm{count}{\textrm{s}}_{\textrm{CT}26-\textrm{Mlh}1-}-\textrm{count}{\textrm{s}}_{\textrm{CT}26-\textrm{Mlh}1+}\ge 10\ \textrm{and}\ \frac{\textrm{count}{\textrm{s}}_{\textrm{CT}26-\textrm{Mlh}1-}}{\textrm{count}{\textrm{s}}_{\textrm{CT}26-\textrm{Mlh}1+}}\ge 10\right).$$

**Table 2:** List of RNAseq performed in CT26 samples. RNAseq features of CT26 *Mlh1*^*+/+*^ and *Mlh1*^*-/-*^ cell lines before and after *in vivo* growth. The total number of sequenced reads and the number of mapped reads on the reference transcriptome are reported in the table

Sample	Mates	Mapped
CT26 *Mlh1*^*+/+*^	62164033	98.73%
CT26 *Mlh1*^*+/+*^ post BALB/c M3	61712172	98.20%
CT26 *Mlh1*^*+/+*^post NOD-SCID M2	59494801	98.72%
CT26 *Mlh1*^*-/-*^	53045412	98.75%
CT26 *Mlh1*^*-/-*^ post BALB/c M2	57481236	98.92%
CT26 *Mlh1*^*-/-*^ post BALB/c M6	59195273	98.82%
CT26 *Mlh1*^*-/-*^ post BALB/c M7	58457417	98.80%
CT26 *Mlh1*^*-/-*^ post NOD-SCID M5	60813574	98.88%

### MHC-I associated peptide annotation

Peptides identified by matching RNAseq database and the immune-peptidomic pipeline were further inspected to determine the genomic regions from which those peptides originated. For both *Mlh1*^*+/+*^ and *Mlh1*^*-/-*^ peptide list, the original read name, the sequence, and frame of translation were retrieved. Next, the relative positions of the peptides inside the reads were calculated. Fasta files were generated and fed to *blat* [32] to retrieve genomic coordinates of the identified peptides. A score was calculated from the *blat* annotated files as follows: (match + rep. match − mis − match − Q _ gap _ count − T _ gap _ count). For each read, only the best score output was selected, and a BED file was generated with the determined genomic coordinates. The latter BED file was further examined through the *bedtools merge* command using -*d 5 -c 4 -o distinct, count* as parameters [29]. Next, the resulting file was matched with the BED file that includes all the genomic regions previously described using the *bedtools intersect* command [29]. Only uniquely mapped peptides were selected. In case peptide reads were aligned to regions that were not uniquely annotated, the following priorities were assigned to the genomic regions: (1) coding sequence; (2) 5’UTR; (3) 3’UTR; (4) intronic; and (5) extragenic. Next, the annotated peptides were matched with the variant calling files to check the presence of SNVs and indels. Finally, all the peptides were examined combining the information from the canonical transcripts, generated from the UCSC refFlat table, to identify in-frame and out-of-frame peptides. The analysis of targeted MAPs in *Mlh1*^*-/-*^ tumor masses excised from BALB/c mice was performed calculating the log_n_ fold change from pre-injection of RNA read counts + 1.

### In vitro T cell assay

To validate immunogenicity of non-coding peptides, the spleens of tumor-rejecting and naïve mice were surgically resected and reduced to single cell suspension. Splenocytes were stained with fluorochrome labeled anti-CD4, CD8, CD44, and CD62L (Biolegend) and analyzed by flow cytometry. Cells were resuspended in RPMI supplemented with 5% FBS, 1% L-glutamine, 1% penicillin and streptomycin, β-mercaptoethanol, and cultured in standing T25 flasks (30×10^6^ cells in 30 ml) in the presence of MAP (pool 1–3, 1 mM). Peptide pools were prepared as detailed in result section and described in Table 4. After 4 days, cultures were harvested, viable cells were separated through a Ficoll gradient, and counted by trypan blue exclusion. Viable cells were cultured overnight with IL-2 and restimulated with the respective MAP pools. Control unrelated peptides were also used. After 48 h, culture supernatants were recovered, and IFN-γ release was measured by ELISA. Prism was used to plot data and non-parametric Mann–Whitney test was performed to evaluate statistical significance.

## Results

### Characterization of immune-targeted alterations in MMR-proficient and MMR-deficient CRC cells

**Fig. 1:**
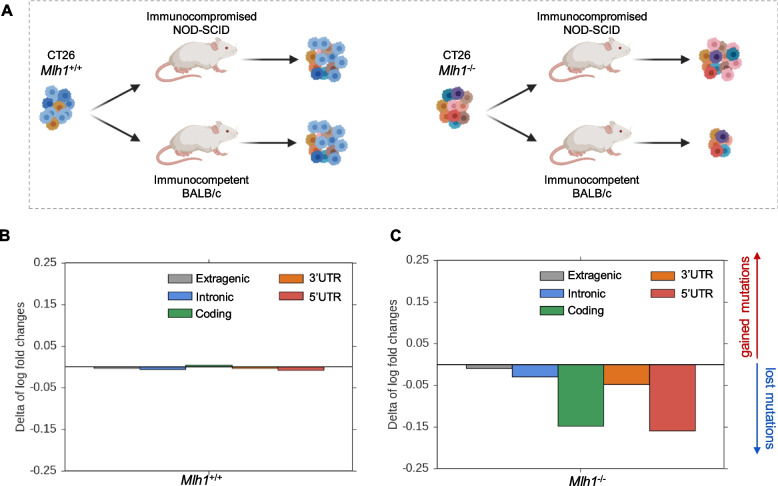
Analysis of mutations targeted in MMR-proficient and MMR-deficient CT26 after injection in immunocompromised and immunocompetent mice. **A** Experimental workflow employed for the analysis of mutations (SNVs and indels) in WGS data of CT26 *Mlh1*^+/+^ and *Mlh1*^-/-^ samples. Briefly, each CT26 clone was inoculated into NOD-SCID (immunocompromised) and BALB/c (immunocompetent) mice 150 days after genome editing. CT26 MMR-proficient and MMR-deficient tumors underwent WGS at the time of injection and after excision from the mice when tumors reached 1200 mm^3^ of volume in NOD-SCID mice. In the case of BALB/c mice, the tumors were excised when they reached volumes of 1100 mm^3^ and 800 mm^3^ for *Mlh1*^*+/+*^ and *Mlh1*^*-/-*^ tumors, respectively. Delta between log fold changes evaluated after injection in immunocompromised and immunocompetent mice in CT26 *Mlh1*^+/+^ (**B**) and CT26 *Mlh1*^-/-^ (**C**). Log fold changes analysis of gained and lost alterations was calculated from CT26 *Mlh1*^+/+^ and *Mlh1*^-/-^ pre-injection data, respectively. The alterations were grouped in regions and normalized per Mb before log fold change calculation

To characterize the landscape of alterations (SNVs and indels), targeted by the immune system when cancer cells were grown *in vivo*, we exploited a previously described syngeneic mouse models [10, 33]. We examined the impact of the immune system on cancer cells by injecting MMR-proficient and MMR-deficient cells into immunocompromised (NOD-SCID) and immunocompetent mice (BALB/c) (Fig. 1A). Tumor cells were subjected to high depth WGS at the day of mouse implantation and at the time of excision, i.e., after 15 days of growth in mice (Table 1). To assess whether and how the genomic profile of *Mlh1*^*+/+*^ and *Mlh1*^*-/-*^ tumor cells evolved in the presence of competent or compromised immune system, we first evaluated the mutational landscape of each sample. More precisely, we calculated the number of SNVs and indels per Mb that emerged in each distinct genomic region (Additional file 1: Fig. S1A and Fig. S1B; Additional file 2: Table 1). We found no differences in the mutational spectrum in MMR-proficient cells (*Mlh1*^*+/+*^) pre- and post-injection in both immunocompetent and immunocompromised mouse models (Additional file 1: Fig. S1A; Additional file 2: Table 1). Overall, these results showed no evidence of immune targeted mutations in MMRp tumors grown in immune competent mice. Conversely, a considerable increase in alterations in all genomic regions of *Mlh1*^*-/-*^ cells were observed compared to *Mlh1*^*+/+*^ cells, particularly in the non-coding areas (Additional file 1: Fig. S1B; Additional file 2: Table 1). Moreover, a trend towards the reduction of mutations per Mb was observed in MMR-deficient cells transplanted in immunocompetent mice compared to cells before the injection and growth in immunocompromised mice. This result prompted us to examine the contribution of the immune system against antigenic mutations; to this end, we calculated the mutational differences of MMR-proficient and MMR-deficient cancer cells that grew in immunocompetent and immunocompromised mice. Specifically, we evaluated the log fold change from preimplantation cells of gain and lost mutations after tumor growth in vivo (Additional file 1: Fig. S1C and Fig. S1D; Additional file 2: Table 2). No differences were observed in gained and lost mutations in *Mlh1*^*+/+*^ clones after injection in immunocompromised and immunocompetent mice (Fig. 1B; Additional file 2: Table 3). On the contrary, a marked reduction (log fold change) was evident in 5’UTR and coding regions of the CT26 *Mlh1*^*-/-*^ genome (Fig. 1C; Additional file 2: Table 3); the 3’UTR and the intronic regions were affected albeit to a lower degree. Overall, these data suggest that alterations occurring in those regions were removed by the activity of the immune system. To further evaluate the impact of the immune system on the mutations, we assessed the ratio between non-silent and silent mutations in WGS of MMRp and MMRd tumors grown in immunocompromised and immunocompetent mice. *Mlh1*^*+/+*^ tumors exhibited a comparable ratio of non-silent to silent mutations after growth in both NOD-SCID and BALB/c mice (Additional file 1: Fig. S2A). In contrast, *Mlh1*^*-/-*^ tumors grown in immunocompetent mice showed a lower ratio of non-silent to silent alterations compared to those observed in tumors grown in immunocompromised mice (Additional file 1: Fig. S2A). This suggests a specific *in vivo* selection process targeting regions that generate potential neoantigens. Moreover, it is known that MMRd tumors exhibit alterations in microsatellite regions, specifically small insertions and deletions [2]. Therefore, we evaluated the status of these regions in the WGS of MMRp and MMRd samples before and after inoculation in immunocompromised and immunocompetent mice. A significant difference in the MSI score was found between *Mlh1*^*-/-*^ and *Mlh1*^*+/+*^ samples (Additional file 1: Fig. S2B). Moreover, *Mlh1*^*-/-*^ tumors showed a higher prevalence of indel alterations in coding and non-coding genomic regions, with many of these alterations occurring in microsatellite regions (Additional file 1: Fig. S2C and Fig. S2D).

Finally, to further investigate immune selection of somatic mutations, we assessed whether gene transcription might influence our results. For this purpose, we aligned DNA and RNA sequencing results and examined the log fold change of gained and lost mutations after *in vivo* tumor growth (Additional file 1: Fig. S3A). We observed a significant reduction in the number of expressed mutations in the UTR and coding regions of *Mlh1*^*-/-*^ cells after injection into immunocompetent mice (Additional file 1: Fig. S3A). Furthermore, we noted *in vivo* selection (lower ratio of expressed non-silent to silent alterations) of expressed mutations in *Mlh1*^*-/-*^ tumors that developed in immunocompetent mice (Additional file 1: Fig. S3B).

### Identification of MHC class I associated peptides

**Fig. 2:**
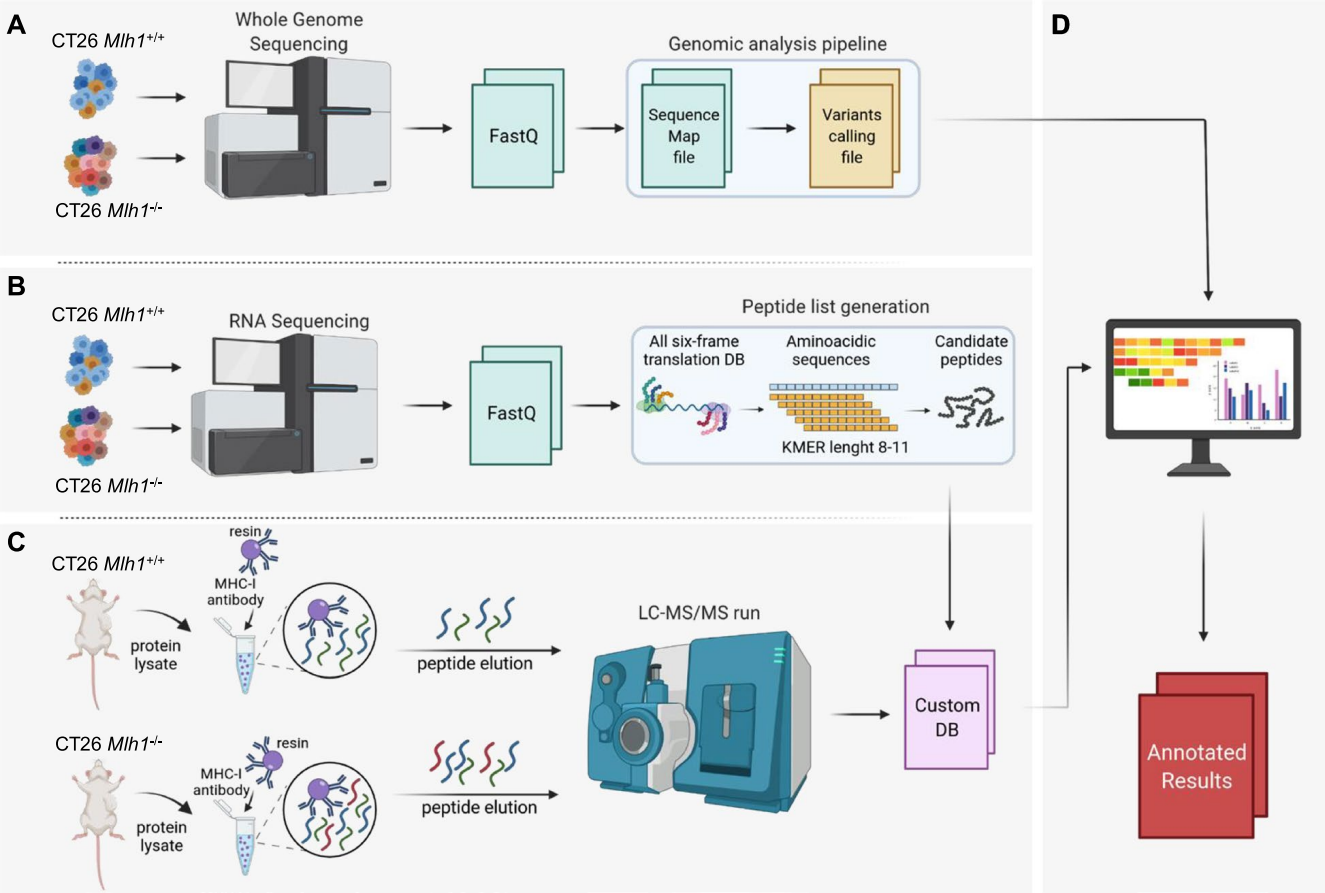
Development of a pipeline for MAP identification. **A** WGS data were generated from CT26 *Mlh1*^+/+^ and *Mlh1*^-/-^ samples and analyzed using IDEA pipeline [28] in order to produce the alignment and variant calling files. **B** RNAseq data were further generated from CT26 *Mlh1*^+/+^ and *Mlh1*^-/-^ cells 150 days after genome editing and after excision from the mice when tumors reached 1200 mm^3^ of volume in NOD-SCID mice. In the case of BALB/c mice, the tumors were excised when they reached volumes of 1100 mm^3^ and 800 mm^3^ for *Mlh1*^*+/+*^ and *Mlh1*^*-/-*^ tumors, respectively. FastQ files were handled to produce the list of all putative peptides present in the transcriptome of each sample. In brief, every transcript sequence in the FastQ files underwent all-six frame translation; then the lists of 8–11 amino acid long peptides were generated using the KMER approach; finally, the peptide lists were compared to select only peptides targeted in tumors excised from immunocompetent mice (see methods). **C** CT26 *Mlh1*^*+/+*^ and *Mlh1*^*-/-*^ tumor masses were explanted from NOD-SCID mice (*n* = 6 per group) and protein extraction was performed. MHC-I was isolated from whole protein lysates through H-2d antibodies conjugated to resin, then peptides were eluted from MHC-I and injected in mass spectrometer. The LC-MS/MS data were then analyzed using MaxQuant. Peptides were searched against the customized DB made of targeted peptides generated by RNAseq data. **D** Sequence results obtained from the immune-peptidomic pipeline were ultimately matched with WGS data to retrieve information about the genomic sources of targeted peptides (see “Methods”)

We reasoned that immunogenicity could be an inherent characteristic of neopeptides independent from the genomic region from which they originated. To identify peptides with an immunogenic value, we therefore considered all genomic regions (coding and non-coding). We assumed that these regions should be transcribed, translated, and further processed to be presented as neoantigens on the cell surface, allowing the generation of neopeptides from genomic non-coding areas. We hypothesized that the non-coding regions selected by the immune system encompassed unconventional MAPs induced by inactivation of the MMR pathway. To test this hypothesis, we developed a comprehensive neoantigen identification pipeline integrating whole genome, RNA sequencing, and an immune-peptidomic profiling through mass spectrometry analysis (Fig. 2). As mentioned above, at first, we performed WGS on *Mlh1*^*+/+*^ and *Mlh1*^*-/-*^ before and after growth in BALB/c and NOD-SCID mice (Fig. 1A) generating a list of SNVs and indels. Then, RNA extracted from the same samples was also sequenced (Table 2). However, the RNAseq FastQ files were not aligned to the reference transcriptome (in contrast to procedures previously performed for the genomic pipeline); instead, the raw data were used to create two different databases (see the following paragraph) containing all the peptides that could originate from the transcripts of both the MMR-proficient and MMR-deficient cancer cells (Fig. 2B). Ultimately, we applied the immune-peptidomic pipeline to unveil the antigenic profile presented by the MHC class I on the surface of *Mlh1*^*+/+*^ and *Mlh1*^*-/-*^ cells (Fig. 2C). Briefly, we performed MHC-I immunoprecipitation on protein lysates of both MMR-proficient and MMR-deficient tumors grown in immunocompromised animals. Next, peptides eluted from the MHC-I molecules were analyzed by LC-MS/MS and searched against a customized reference databases that contained all putative peptide sequences selected by RNAseq analysis. The specificity of each peptide was verified by cross-check of the *Mlh1*^*+/+*^ and *Mlh1*^*-/-*^ databases on both samples that is *Mlh1*^*+/+*^ eluted peptides against the *Mlh1*^*-/-*^ specific database and vice versa. Through this approach, we selected only *Mlh1*^*-/-*^ exclusive peptides (*Mlh1*^*-/-*^ specific database). Finally, we merged the results from the mass spectrometry analysis and the WGS analysis to characterize the mutational status and annotate the genomic origin of MAPs (Fig. 2D).

### Identification of immune-targeted peptides in *Mlh1*^*+/+*^ and *Mlh1*^-/-^ tumor cells

**Fig. 3:**
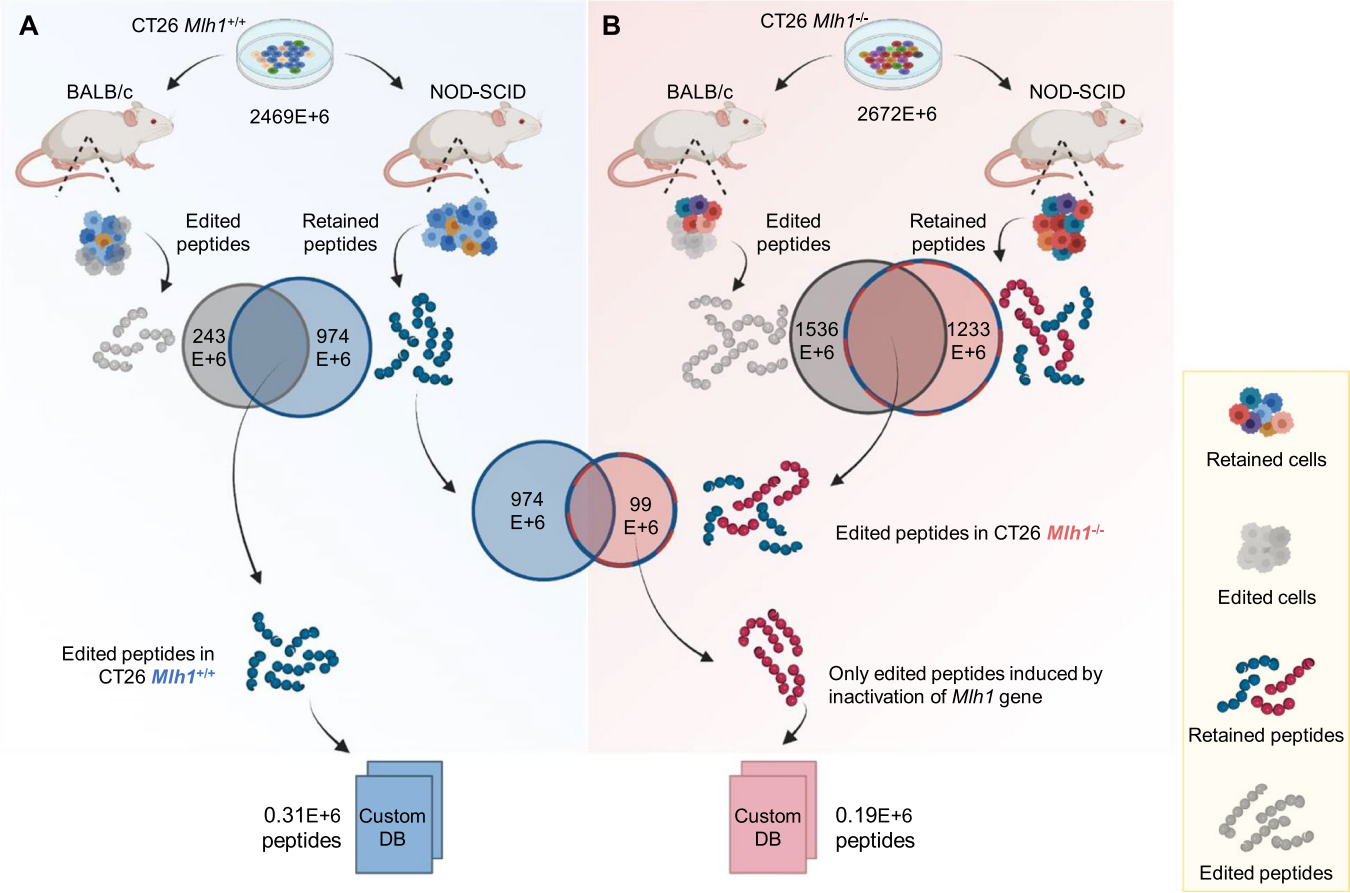
Identification of targeted MAPs in *Mlh1*^+/+^ and *Mlh1*^*-/-*^ tumor cells. **A** The peptide list generated from RNAseq analysis of CT26 *Mlh1*^*+/+*^ cells grown *in vitro* was compared to the corresponding lists obtained after tumor growth in mice (see Table 2). Thus, peptides lost after injection in CT26 *Mlh1*^*+/+*^ post BALB/c M3 mouse and retrieved after inoculation in CT26 *Mlh1*^*+/+*^ post NOD-SCID M2 mouse were selected. The overlap of these two peptide datasets generated the database of CT26 *Mlh1*^*+/+*^ targeted peptides. **B** Peptide lists generated from RNAseq analysis in *Mlh1*^*-/-*^ samples before and after *in vivo* growth were compared (see Table 2). This allowed the identification of peptides lost after injection in CT26 *Mlh1*^*-/-*^ post BALB/c M2, M6, and M7 mice but maintained in CT26 *Mlh1*^*-/-*^ post NOD-SCID M5 mouse. The overlap of these two datasets generated a list of peptides from which specific CT26 *Mlh1*^*+/+*^ sequences were removed. The latter list created the targeted peptides database specific to CT26 *Mlh1*^*-/-*^

To generate a peptide database for mass spectrometry analysis, we exploited both immunocompromised and immunocompetent mice. This strategy enabled us to selectively choose peptides that were putatively targeted by a functional immune system. Before applying the immune-peptidomic pipeline (Fig. 2C), we verified the cell surface levels of MHC class I in both MMR-proficient and MMR-deficient cell models (Additional file 1: Fig. S4A and Fig. S4B). The RNAseq analysis of tumor cells, from which we inferred the peptide sequences, revealed over 2,469 million possible amino acid sequences from *Mlh1*^*+/+*^ transcripts (Fig. 3A). To specifically select peptides targetable by the immune system, we identified the translated sequences that were lost after *Mlh1*^*+/+*^ cells were grown in immunocompetent animals (and therefore targeted by the immune system) and retained after injecting *Mlh1*^*+/+*^ cells into immunocompromised mice. Importantly, we considered sequences retained in immunocompromised mice to exclude those that were lost during *in vivo* growth but were unrelated to immune editing. The combined results generated a list of 305,506 peptides from which a custom database for *Mlh1*^*+/+*^ cells was constructed.

We subsequently applied the same workflow to *Mlh1*^*-/-*^ cells leading to the identification of 99 million sequences that were lost in immunocompetent mice but retained in immunocompromised mice (Fig. 3B). To pinpoint sequences induced solely by the inactivation of MMR machinery, peptides found in *Mlh1*^*-/-*^ cells were excluded if they were also present in their *Mlh1*^*+/+*^ counterpart. This process led to the identification of a total of 193,312 sequences which were identified in the *Mlh1*^*-/-*^ custom database (Fig. 3B).

The overall approach identified 417 peptides specifically present in *Mlh1*^*+/+*^ cells, while 775 peptides were found to be specific of *Mlh1*^*-/-*^ tumors (Table 3).

**Table 3:** List of LC-MS/MS run results in CT26 samples. Number of MAPs identified in *Mlh1*^*+/+*^ and *Mlh1*^*-/-*^ tumor masses excised from NOD-SCID mice. The spectra results were matched with the custom and UniProt mouse databases

Sample	*Mlh1*^+/+^ or *Mlh1*^-/-^ custom database	Uniprot mouse database
CT26 *Mlh1*^*+/+*^ post NOD-SCID	417	234
CT26 *Mlh1*^*-/-*^ post NOD-SCID	775	362

### Classification of MHC class I associated peptides in *Mlh1*^*+/+*^ and *Mlh1*^*-/-*^ murine CRC cell lines

To identify the genomic regions from which immunogenic MAPs originated, we exploited WGS data. Firstly, we investigated peptides originating from translated DNA sequences in *Mlh1*^*+/+*^ and *Mlh1*^*-/-*^ cells. Subsequently, we conducted a refining alignment of peptides derived from specific reads and annotated them on the mouse genome. Next, each genomic region was assigned to *coding, 3’UTR, 5’UTR, intronic, or extragenic* labels. Furthermore, based on the open reading frame of the sequences and the canonical isoforms annotated in the mouse transcriptome, each peptide was further annotated as either *in-frame* and *out-of-frame*. In total, we were able to confidently annotate 396 (95%) and 665 (86%) MAPs in *Mlh1*^*+/+*^ and *Mlh1*^*-/-*^ cells, respectively, that were lost after growth in immunocompetent mice (Additional file 1: Fig. S5A; Additional file 2: Table 4 and Table 5). Interestingly, our results showed that most MAPs targeted by the immune system were derived from non-coding regions (Additional file 1: Fig. S5B; Additional file 2: Table 4 and Table 5). More specifically, the majority of them were classified as non-canonical, since many MAPs, albeit originated from coding regions, showed *out-of-frame* translations in both MMR-proficient and MMR-deficient cells. We took advantage of the variant calling files obtained from the genomic analysis pipeline to study which type of mutations (SNVs or indels) affected the MAPs. Notably, mMAPs were most abundant in *Mlh1*^*-/-*^ cells and were mainly located in coding and UTR regions (Additional file 1: Fig. S5A and Fig. S5B; Additional file 2: Table 4 and Table 5). On the contrary, *Mlh1*^*+/+*^ cells displayed only few mutated MAPs.

**Fig. 4:**
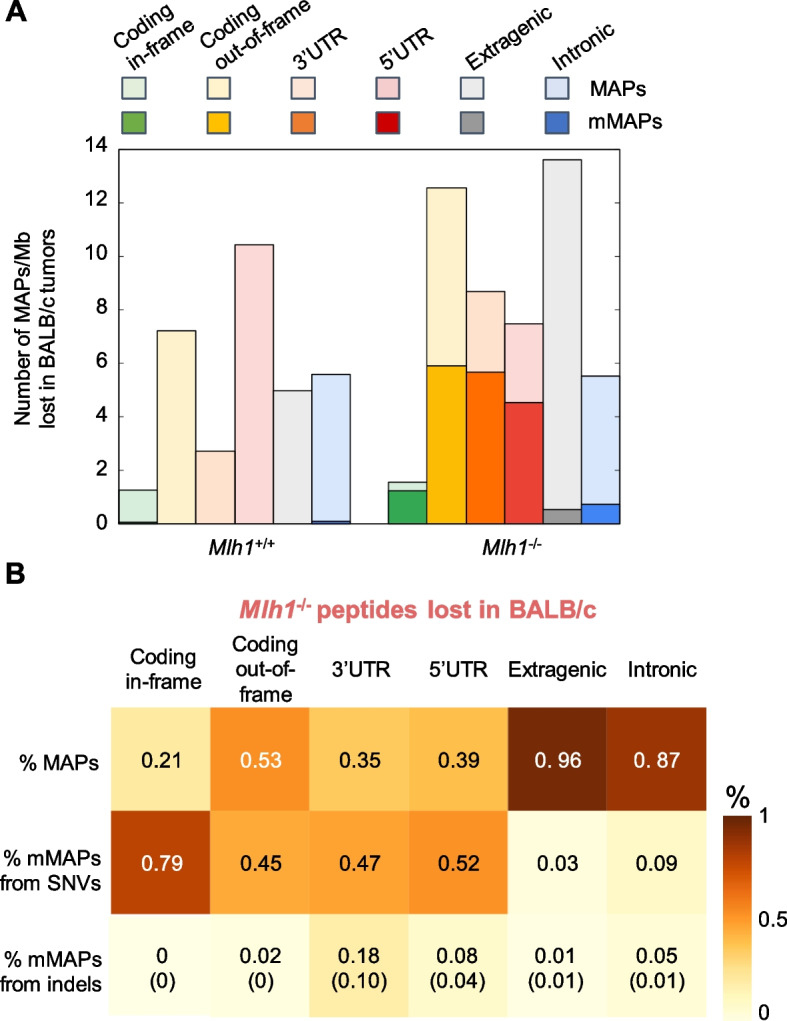
Characterization of targeted non-canonical MAPs and mMAPs in *Mlh1*^+/+^ and *Mlh1*^*-/-*^ tumor cells. **A** The numbers of annotated MAPs were normalized (per Mb) in CT26 *Mlh1*^*+/+*^ and *Mlh1*^*-/-*^ samples and are reported in light colors. mMAPs are highlighted in solid colors. **B** Percentage of mutated and wild-type *Mlh1*^*-/-*^ MAPs. Mutated MAPs originated from indels were further labeled with microsatellite information (data in brackets)

We considered that the polyA capture technique of RNA molecules for the subsequent RNAseq analysis could have influenced the prevalence of MAPs in specific regions since they were better represented in the transcriptome. For this reason, we normalized the peptide classification according to RNAseq coverage data in each region (Additional file 1: Fig. S6A and Fig. S6B; Additional file 2: Table 6). This analysis showed a higher prevalence of 5’UTR-derived MAPs per Mb in both *Mlh1*^*+/+*^ and *Mlh1*^*-/-*^ models, while the overall trend of all other regions did not change (Fig. 4A; Additional file 2: Table 4 and Table 5). Moreover, a deep characterization of mMAPs resulted in greater prevalence of alterations (SNVs and indels) in coding and UTR regions as compared to intronic and intergenic ones (Fig. 4B).

To assess the reliability of our workflow, we decided to apply the immune-peptidomic pipeline against the UniProt mouse database (Additional file 1: Fig. S7A). We identified 171 mouse MAPs in common to both MMR-proficient and MMR-deficient cells, while 63 and 191 exclusive MAPs were found in the *Mlh1*^*+/+*^ and *Mlh1*^*-/-*^ clones, respectively (Table 3, Additional file 1: Fig. S7B). As expected, the sequences present in the mouse canonical database were classified as coding. To further corroborate our findings, we determined the change in the expression level of each putatively targeted MAP in *Mlh1*^*-/-*^ tumors grown in immunocompetent mice, taking into consideration the biological variability of expression across different animals. To this end, first, we evaluated the abundance of RNA sequences supporting the peptide calls in CT26 *Mlh1*^*-/-*^ before- and after-growth in immunocompetent animals (Additional file 1: Fig. S8). We found 248 MAPs whose expression was completely lost in at least two out of three tumors grown in immunocompetent mice (Additional file 1: Fig. S8). Then we calculated the log fold change (Additional file 1: Fig. S9A). Notably, mutated MAPs exhibited a lower expression in *Mlh1*^*-/-*^ cells grown in mice as compared to *wild-type* MAPs, suggesting that those sequences were likely targeted by the immune system of the host. Indeed, fold change analysis revealed a statistically significant reduction of mMAP transcripts (grouped in coding and non-coding) compared to *wild-type* MAPs in *Mlh1*^*-/-*^ cells grown in immunocompetent mice (Additional file 1: Fig. S9B).

### Immunogenicity analysis of non-canonical MAPs in *Mlh1*^*-/-*^ CRC cells

**Fig. 5:**
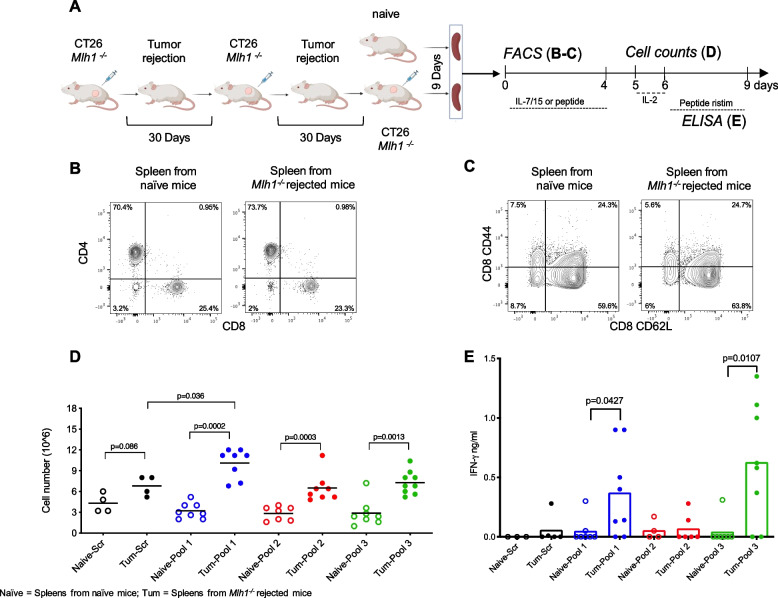
Non-canonical MAP-specific T cells in mice rejecting *Mlh1*^*-/-*^ tumors. **A** Immunocompetent mice (BALB/c) were injected with 5X10^5^ *Mlh1*^*-/-*^ tumor cells per mouse. Upon rejection, mice were re-challenged twice with *Mlh1*^*-/-*^ tumor cells (30 days after the previous injection). Nine days after the last injection, mice were sacrificed, and the spleen were surgically resected. Naïve mice were used as control. **B** Representative events of viable CD4^+^ and CD8^+^ T cells are shown in naïve mice and tumor rejected mice. **C** Single cells were stained with anti-CD4, CD8, CD44, and CD62L mAb and analyzed by FACS. Data depicts naïve and memory markers of viable CD8^+^T cells. **D** Splenocytes were cultured with or without synthesized MAP peptides for 4 days. Viable cells were separated on Ficoll gradients and counted. Total counts are depicted. **E** After an overnight incubation in IL-2, T cells, from naive and tumor-vaccinated mice, were either restimulated or not with the indicated peptide pools. A scramble peptide (Ctrl) served as control. The supernatant was collected after 48 h to quantify IFN-γ. The data show the release of IFN-γ from splenocytes of individual mice pulsed with scramble or peptide pools. The values of IFN-γ from unpulsed splenocytes were subtracted from the values represented in the figure. *p*-values were calculated by Mann–Whitney non-parametric test

Next, we asked whether T cells capable of recognizing non-canonical MAPs-specific might be identifiable in immunocompetent mice rejecting *Mlh1*^*-/-*^ tumors. To restrict the number of candidates, we applied the following filters: (a) MAPs that were present in the RNAseq of CT26 *Mlh1*^*-/-*^ cells; (b) MAPs not found in all three tumors grown in immunocompetent mice (Additional file 1: Fig. S8); (c) MAPs generated from atypical translational events; and (d) MAPs with high expression levels, MS intensity and allele frequency (in case of mutations). We obtained a list of 20 candidates, the corresponding peptides were synthetized and grouped in three pools for *in vitro* testing (Table 4). Then, we challenged mice with *Mlh1*^*-/-*^ tumor cells (5×10^5^ cells/mouse). After 15 days from the injection, 10 out of 20 mice were tumor free. Tumor-rejecting mice were rechallenged with tumor cells after an additional 2 weeks. Nine days after the last tumor cell challenge, mice were sacrificed, and the spleen collected to investigate whether peptide-specific T cell responses could be identified (Fig. 5A). T cell representation and phenotype was first analyzed by FACS. We found that naïve and tumor-rejecting mice contained comparable frequencies of CD4^+^ and CD8^+^ T cells with a similar distribution of naïve and memory subsets (Fig. 5B and C). Splenocytes were then stimulated with peptide pools for 4 days. Of note, peptide-driven cultures of tumor-rejecting mice revealed total cell counts higher than those found in splenocyte cultures derived from naïve mice (Fig. 5D). Differences were mostly found when pool 1 and pool 3 were used (Fig. 5D). To test the presence of peptide-specific memory T cells, splenocytes were further stimulated for 48 h with control peptides or MAP peptide pools and IFN-γ production investigated in culture supernatants. Results reported in Fig. 5E indicate higher IFN-γ production in pool 1 and pool 3 restimulated cultures, compared to control (Fig. 5E). Together, these data identify non-canonical MAP-specific T cells in the spleen of mice rejecting *Mlh1*^*-/-*^ tumors, providing functional evidence of the immunogenicity of newly identified antigens.

**Table 4:** List of MAPs selected for ex vivo immunoassay. Annotated list of 20 peptides identified in *Mlh1*^*-/-*^ cells

Peptides	Genomic coordinates of pep seq	Region	#Reads	Alterations	Canonical	%AF	MS replicates	MS intensity	Gene FPKM	Pool
NKKNEGWLSKP	chr19:40559969-40560003	intron	10	WT	Non-canonical	100	1	NA	50.32	1
EMKPFIPI	chrX:104994035-104994060	extragenic	10	WT	Non-canonical	100	1	11750000	NA	1
FRGLTETTSSL	chr8:34821561-34821595	extragenic	12	WT	Non-canonical	100	1	NA	NA	1
FLRRCCLARYE	chrX:142351611-142351618 chrX:142390305-142390331	utr5	10	WT	Non-canonical	100	1	NA	26.06	1
LKIAWLREICL	chr9:14546274-14546308	utr3	16	SNV	Non-canonical	15.9574	1	NA	23.57	1
YRRFSFEG	chr7:118853174-118853202	cds	12	SNV	Non-canonical	14.4144	2	15288667	14.72	2
VKMPDAPR	chr12:105662622-105662648	cds	11	SNV	Non-canonical	18.5185	1	16421000	5.59	2
CASARCSPCLT	chr2:34870760-34870794	cds	15	SNV	Non-canonical	12.973	1	NA	39.25	2
AEKLKRAAEKD	chrX:136085159-136085193	utr5	12	WT	Non-canonical	100	1	NA	5.46	2
HKKPQPVPRAH	chr6:120482825-120482859	utr3	15	SNV	Non-canonical	15.8273	1	NA	9.42	2
RQWERTVKNKQ	chr5:67621541-67621575	utr3	11	WT	Non-canonical	100	1	NA	1.47	2
ELRLRHIAWAL	chr11:96712585-96712619	intron	14	WT	Non-canonical	100	1	NA	0.82	2
ITINPIKSMLF	chr16:30341231-30341265	cds	17	WT	Non-canonical	100	1	NA	43.64	3
IFPSIGDLALS	chr14:101918019-101918044 chr14:101918612-101918620	cds	14	WT	Non-canonical	100	1	NA	78.13	3
PSAFILIRKLG	chr7:45013466-45013500	cds	12	SNV	Non-canonical	16.7883	1	NA	38.55	3
PVVCKSQLAHG	chr1:164050176-164050187 chr1:164050644-164050666	cds	21	WT	Non-canonical	100	1	NA	20.21	3
VLVMFLLGKLP	chr4:82804025-82804058	utr3	18	WT	Non-canonical	100	1	NA	17.02	3
EPHSVTELKLE	chrX:136079187-136079221	intron	11	WT	Non-canonical	100	1	NA	5.46	3
SAVWTDLKMTP	chr1:153453947-153453981	extragenic	10	WT	Non-canonical	100	1	NA	NA	3
KCFSVISLYFE	chr5:67732187-67732221	cds	10	WT	Non-canonical	100	1	NA	1.47	3

## Discussion

Although molecular defects in the MMR machinery have been associated to an aggressive phenotype that leads cancer to rapid molecular evolution and uncontrolled dissemination, considerable evidence has highlighted how this is a double-edged sword for tumor cells [34]. We previously showed that MMRd tumors trigger a remarkable immune response owing to their high neoantigen burden [10]. We reported that a higher CD8^+^ T-cell infiltration was present in the tumor microenvironment alongside a high number of distinct T cell receptor rearrangements in blood of tumor bearing mice [10]. While the contribution of coding DNA to the neoantigen repertoire of tumors has been analyzed by several groups [8, 12, 35], it is currently unknown whether and to what extent the non-canonical neoantigen landscape, sometimes referred to as the “dark” side of the genome (i.e., the non-coding part), plays a role in the immunogenic features of MMRd tumors. Laumont and colleagues demonstrated that in murine cancer cell lines and in human primary tumors—bearing different haplotypes—90% of the identified tumor-associated antigens originated from non-canonical regions [20, 21]. In addition, Chen and colleagues recently demonstrated that 240 non-canonical peptides derived from upstream open reading frames located in the 5’UTR and long non-coding RNAs of extragenic DNA were presented by the HLA of human tumor cell lines [19]. This new knowledge would not have been generated if the tumor associated antigens were identified by standard exome-based approaches. A recent work by Cleyle and colleagues demonstrated the presence of MAPs originating from non-coding regions in MSS and MSI CRCs [36]. However, it remains largely unknow whether tumor specific antigens loaded on the MHC class I can trigger an immune response [36]. Current human models do not allow to determine whether MAPs can be targeted by the immune system of the host. To bridge this gap, we studied the contribution of tumor-associated antigens originating from non-canonical genome in a MMRd murine cell line and its isogenic MMRp counterpart. We performed high depth WGS of *Mlh1*^*+/+*^ and *Mlh1*^*-/-*^ isogenic CT26 cells and observed an increase of the mutational burden associated with mismatch repair inactivation across all the genome and particularly in the extragenic and intronic portions. Then, we injected both isogenic cell lines into immunocompromised and immunocompetent mice and used WGS to establish the mutational burden from SNVs and indels. To identify the genome areas poorly represented after *in vivo* growth which we considered as evidence that immune editing had occurred. We found a reduction of alterations across the genome in both mouse strains, this is likely due to technical procedures as previously described [37]. However, we interestingly found that among the alterations maintained in immunocompromised mice, those in coding and 5’UTR were lost in *Mlh1*^*-/*-^ tumors grown in immunocompetent mice. Next, to identify peptides loaded on the MHC class I complex, we built an immune-peptidomic pipeline combining RNA sequencing and mass spectrometry technology. Since the identification of amino acid sequences bound to the MHC class I complex requires a list of candidate peptides to be matched with, we assembled two specific RNA databases with all the peptide sequences potentially generated by the transcripts of *Mlh1*^*+/+*^ and *Mlh1*^*-/- *^CT26 cell lines. We specifically selected peptides retained in tumors grown in immunocompromised mice and at the same time lost in immunocompetent mice. This approach led to identify hundreds of MAPs in *Mlh1*^*+/+*^ and *Mlh1*^*-/-*^ CT26 cells. Finally, to characterize the mutational status and the areas of the genome from which MAPs originated, the sequences obtained from the immune-peptidomic pipeline were combined with the WGS data. Our results show that in both *Mlh1*^*+/+*^ and *Mlh1*^*-/-*^ cells, most of the MAPs lost in immunocompetent mice originated from non-coding DNA portions in accordance with previous studies [21, 38]. Furthermore, the non-mutated MAPs targeted by the immune system in *Mlh1*^*-/-*^ predominantly originated from non-coding regions whereas mutant MAPs derived primarily from the UTR and coding regions. To define the relative contribution in terms of immunogenicity between non-mutated MAPs and mMAPs, we first calculated their representativeness by the number of supported RNA sequences; then, we calculated the fold change between the number of MAPs lost in tumor cells after *in vivo* growth and those previously present at the day of injection. Interestingly, we observed that the mMAPs were immune targeted more than the non-mutated sequences. The latter observation stems from the characterization of SNVs and indels, which are considered the most abundant events in MMRd cancers [2]. We cannot exclude the possibility that other types of alterations, such as unconventional splice junctions and posttranslational events may also occur in MMR-deficient cancers and contribute to the immune reactivity of these tumors [39–41]. Finally, to directly challenge the immunogenicity of the non-canonical MAPs identified by our computational approach, we selected a representative subset to generate three pools to be used to restimulate T cells in vitro. We found that two of the three pools analyzed were able to trigger T cell expansion *in vitro*, among which MAP-specific T cells capable of specific IFN-γ release. Although further studies will be needed to identify relative representation of T cells specific for individual peptides, and to determine whether immunodominance might occur, the finding that peptide-specific T cell responses are enriched in tumor-rejecting mice support acquired antigenicity and immunogenicity of *Mlh1*^*-/-*^ tumors. We acknowledge that certain neoantigens may evade the immune system pressure through downregulation of their expression levels [42]. Although our experimental design enabled the comprehensive characterization of multiple MAPs that were eliminated in the presence of a competent immune system, we cannot disregard the possibility of such an occurrence in our model. Nevertheless, our objective was to identify unconventional MAPs within a MMRd system and validate their immune effectiveness.

In conclusion, we provide functional evidence that non-coding DNA sequences, which represent 98% of the genome, can contribute to the immunogenic features of MMRd tumors. Additionally, our findings support the relevance of a thorough characterization of tumor samples at the genomic levels including the often overlooked “dark” portion of the genome.

An elevated mutational burden is currently considered a promising independent prognostic biomarker for MMRd cancer [43, 44]. In addition, many CRC patients display a low TMB and are not considered good candidates for ICB therapies. However, most TMB analyses are performed by WES or by custom panels that include a limited number of genes or a portion of them. Accordingly, in most of the studies, the extragenic areas of the genome, that are the vast majority of the entire DNA sequence, are not included in the TMB evaluation. This is noteworthy in light of recent findings from Frigola and colleagues that demonstrate how generation of mutations occurs at lower levels in coding than in the non-coding regions [45]. Notably, they showed that mismatches in exonic DNA are repaired by MMR more efficiently than in their intronic counterparts. Therefore, non-coding regions could accumulate more alterations during tumor evolution as a result of distinct DNA repair efficiency. These findings lead us to speculate that [1] the evaluation of the extragenic part of the genome could improve the definition of the tumor mutational landscape and [2] in MMRd tumors, the contribution of extragenic alterations to generating an immune response could be more impactful than the intragenic part, considering the diverse level of fidelity between intra- and extragenic DNA repair pathways.

## Conclusions

Our study highlights the role of non-canonical MAPs in triggering an immune response in MMRd mouse models. We point out that 5’UTR and 3’UTR regions are a source of mutated peptides that can be loaded on the MHC class I complex. Furthermore, we found that those mMAPs are lost after the growth of MMRd CRC tumors in immunocompetent mice, whereas they are preserved in immunocompromised mice. Finally, we validated antigenicity and immunogenicity of a representative selection of *Mlh1*^*-/-*^ MAPs by the identification of MAP-specific T cell responses in *in vitro* T cell assays. These results suggest that non-canonical MAPs can indeed trigger unique immune responses contributing to MMRd tumor immune editing and to the control of tumor growth.

Overall, we provide a proof-of-concept that in MMRd tumors, non-canonical translational events across the entire genome, i.e., translation of non-coding and out-of-frame coding regions, can effectively contribute to the immunogenic properties of these tumor types and should be evaluated in precision medicine approaches for cancer patients that are being considered for immune-based therapies.

### Supplementary Information

**Additional file 1.** Supplementary figures S1-S9.

**Additional file 2: **TMB and MAPs characterization in CT26 *Mlh1*^*+/+*^ and *Mlh1*^*-/-*^*.*

## Data Availability

All data generated or analyzed during this study are included in the Additional file 2. RNA and whole genome sequencing data are available in the European Nucleotide Archive (ENA) repository with the following accession code PRJEB58630 (https://www.ebi.ac.uk/ena/browser/view/PRJEB58630) [46].

## Abbreviations

MMR: Mismatch repair
MMRp: Mismatch repair proficient
MMRd: Mismatch repair deficient
SNV: Single-nucleotide variant
Indel: Insertion and deletion
CRC: Colorectal cancer
MSS: Microsatellite stable
MSI: Microsatellite unstable
MHC: Major histocompatibility complex
ICB: Immune checkpoint blockade
FDA: Food and Drug Administration
UTR: Untranslated region
MAP: MHC class I associated peptide
mMAP: Mutated MHC class I associated peptide
WGS: Whole genome sequencing
RNAseq: RNA sequencing
FDR: False discovery rate
gDNA: Genomic DNA
NGS: Next-generation sequencing
BED: Browser extensible data
UCSC: University of California Santa Cruz
ncRNA: Non-coding RNA
TMB: Tumor mutational burden
Mb: Megabase
RIN: RNA integrity number
BAM: Binary alignment and map
TPM: Transcripts per million
GR: Genomic region
PI: Principal investigator
BCA: Bicinchoninic acid assay
TFA: Trifluoracetic acid
DDA: Data-dependent acquisition
IL-2: Interleukin 2
IFN: Interferon
ELISA: Enzyme linked immunosorbent assay
HLA: Human leukocyte antigen
