# Supplementary material for: Non-canonical antigens are the largest fraction of peptides presented by MHC class I in mismatch repair deficient murine colorectal cancer
Source: Genome Med. 2024 Jan 19;16:15. doi: 10.1186/s13073-023-01275-3 (PMC10797964; doi:10.1186/s13073-023-01275-3)
Supplement: Supplementary file 1 — Additional file 1. Supplementary figures S1-S9. [file 13073_2023_1275_MOESM1_ESM.pdf]

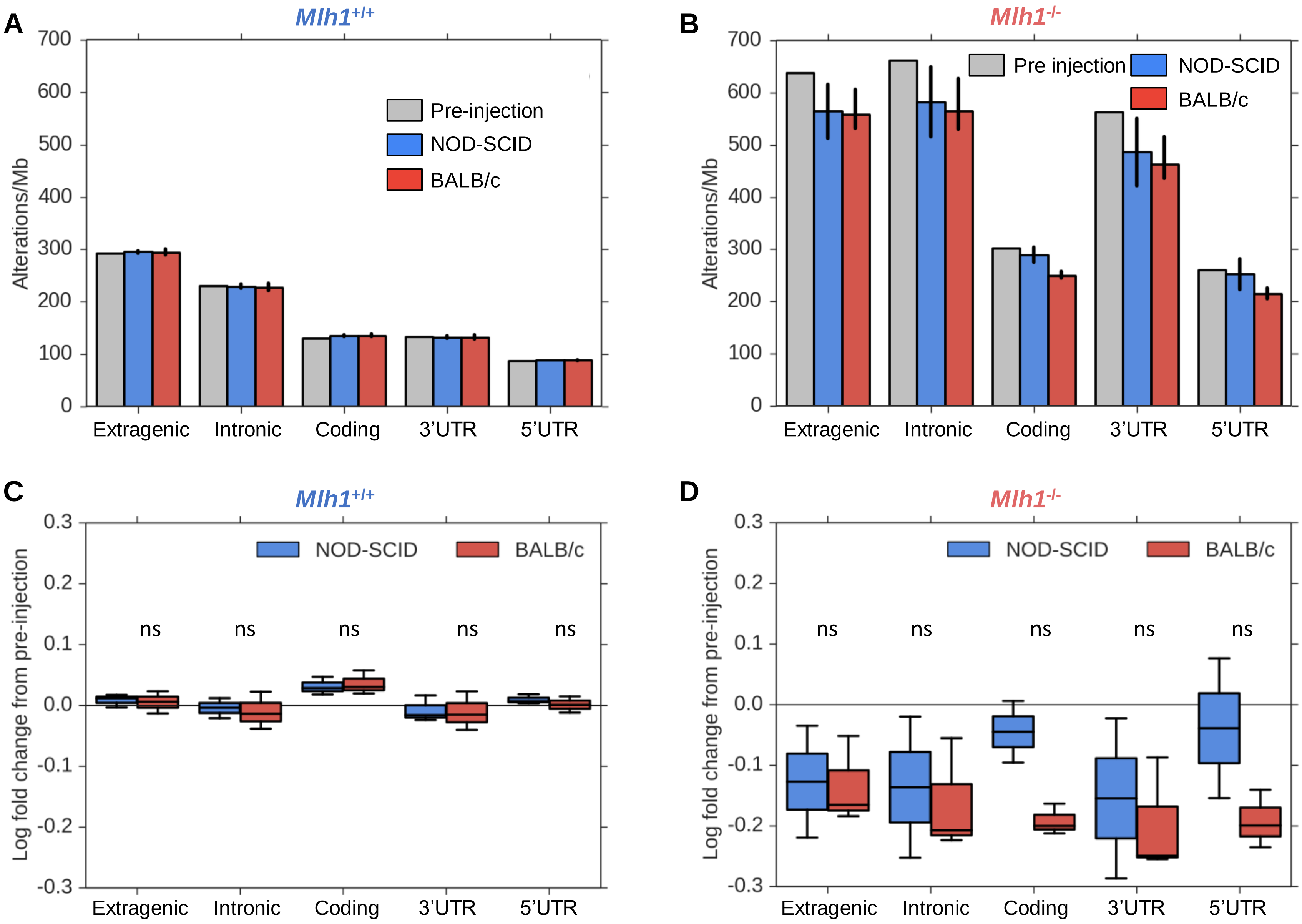

Fig S1: Characterization of alterations in CT26 samples before and after injection in immunocompromised and -competent mice. Number of alterations (SNVs and Indels) per Mb calculated in every genomic region of CT26 *Mlh1*$^{+/+}$ **(A)** and CT26 *Mlh1*$^{-/-}$ **(B)** pre- and post-growth in immunocompromised and -competent mice calculated by WGS. The alterations were grouped in regions and normalized per Mb. Log fold change analysis of gained and lost alterations from pre-injection evaluated after tumor growth of CT26 *Mlh1*$^{+/+}$ **(C)** and CT26 *Mlh1*$^{-/-}$ **(D)** in immunocompromised and -competent mice. The alterations were grouped in regions and normalized per Mb before log fold change calculation. (Mann-Whitney U test: ns non-significant)

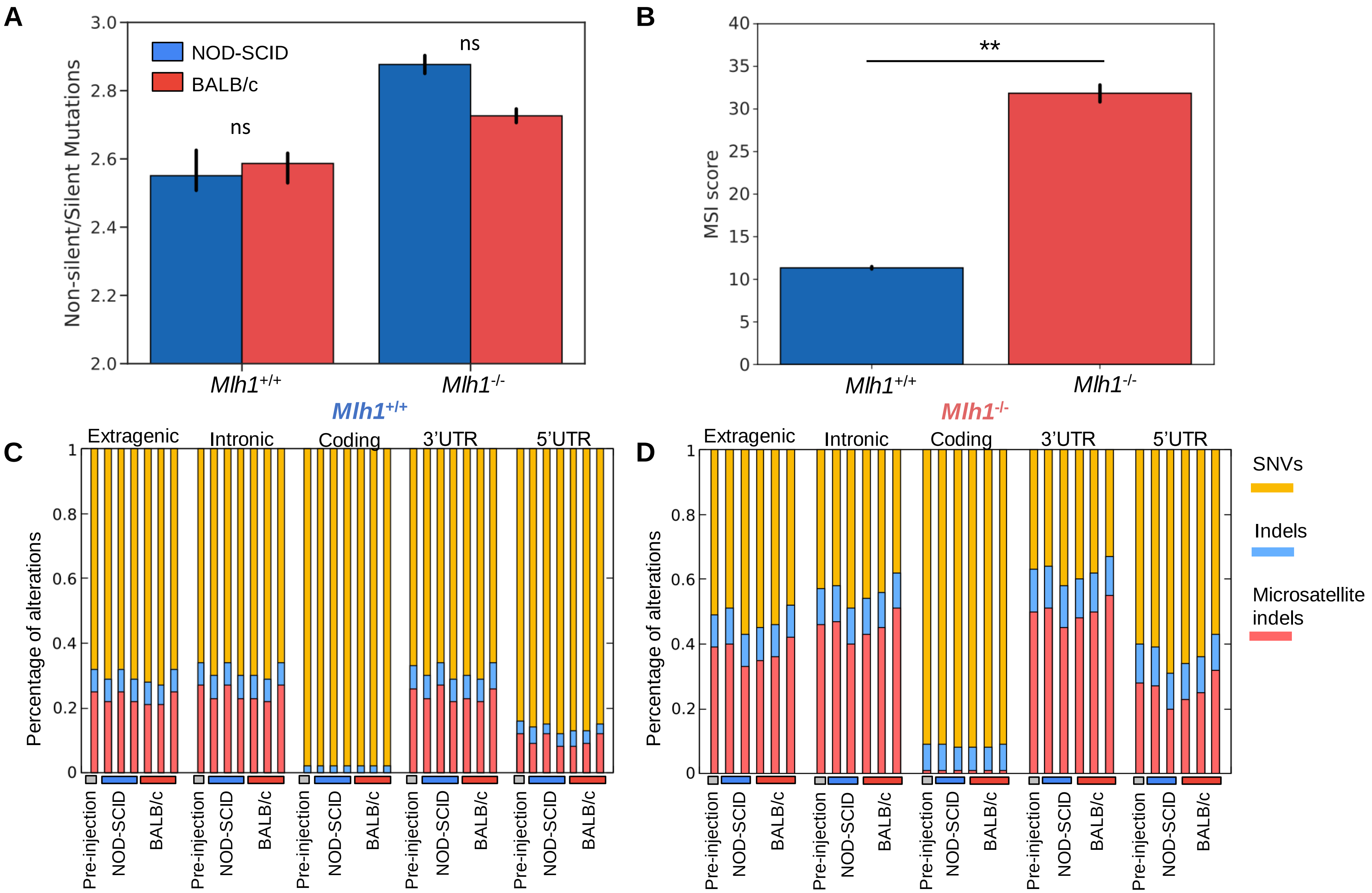

Fig S2: Non-silent over silent mutations and indels characterization in *Mlh1*$^{+/+}$ and *Mlh1*$^{-/-}$ tumors grown in mice. (A) Non-silent/Silent mutations ratio in *Mlh1*$^{+/+}$ and *Mlh1*$^{-/-}$ tumors evaluated in WGS data. Coding mutations acquired in *Mlh1*$^{+/+}$ and *Mlh1*$^{-/-}$ tumors were classified as silent and non-silent according to the aminoacidic changes. **(B)** MSI score of *Mlh1*$^{+/+}$ and *Mlh1*$^{-/-}$ WGS samples. MSI scoring shows the percentage of mutated microsatellite regions across the genome of MMRp and MMRd mouse models (see method section). The percentage of mutations that represents the TMB of *Mlh1*$^{+/+}$ **(C)** and *Mlh1*$^{-/-}$ **(D)** samples in all genomic region is shown. SNV and indel percentages were deciphered from the TMB analysis performed in our study. Microsatellite indels were calculated by matching the mutated loci defined by the MSIsensor pipeline. (Mann-Whitney U test: ns non-significant; ** p-value < 0.005).

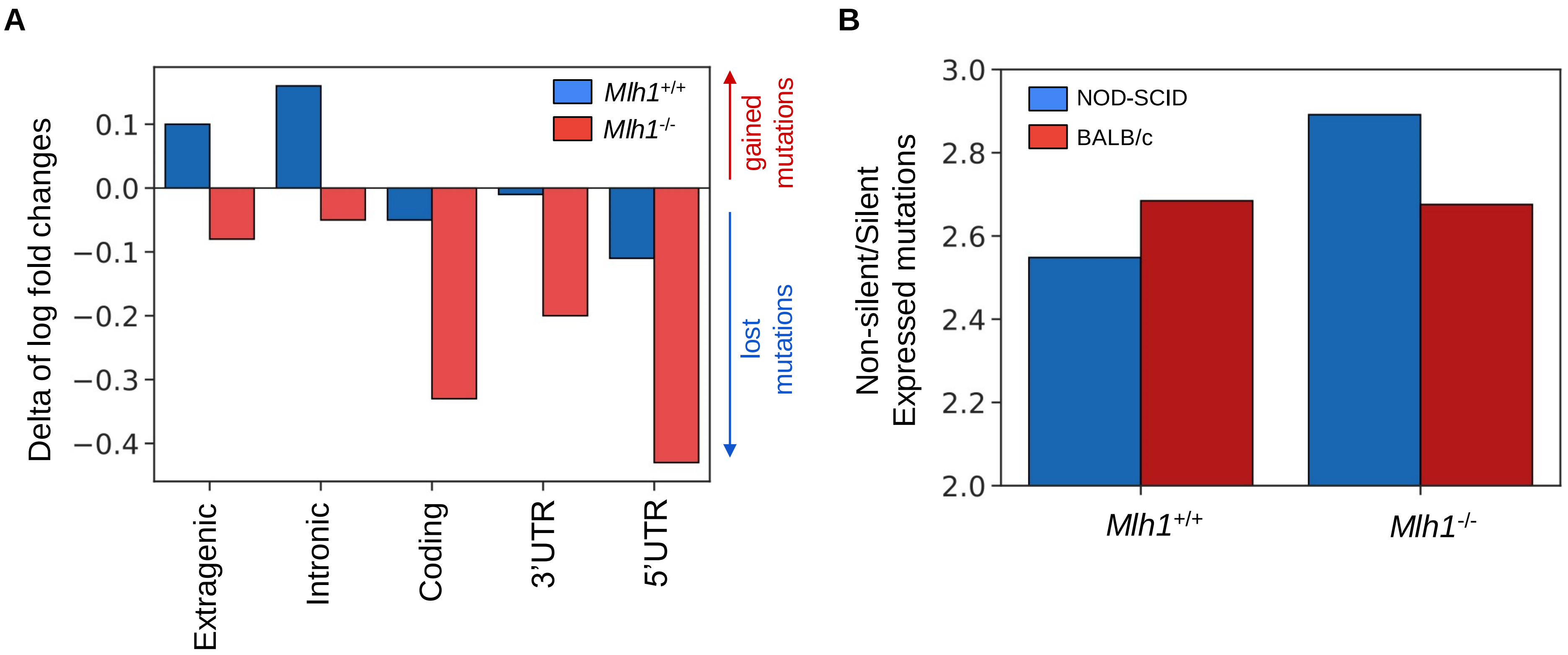

Fig S3: Transcriptomic analysis of gained and lost alterations of *Mlh1*$^{+/+}$ and *Mlh1*$^{-/-}$ tumors after *in vivo* growth. (A) Differences in log fold changes of SNVs between *Mlh1*$^{+/+}$ and *Mlh1*$^{-/-}$ pre-injected cells and their respective tumors grown in immunocompromised and -competent mice. Alterations were grouped by regions and normalized per Mb before calculating the log fold change. **(B)** The ratio of non-silent to silent mutations in *Mlh1*$^{+/+}$ and *Mlh1*$^{-/-}$ tumors as determined from combined WGS and RNAseq data is shown. Coding mutations acquired in *Mlh1*$^{+/+}$ and *Mlh1*$^{-/-}$ tumors were classified as silent and non-silent based on the aminoacidic changes. Non-parametric Mann-Whitney test was performed; however, differences among samples were not statistically significant.

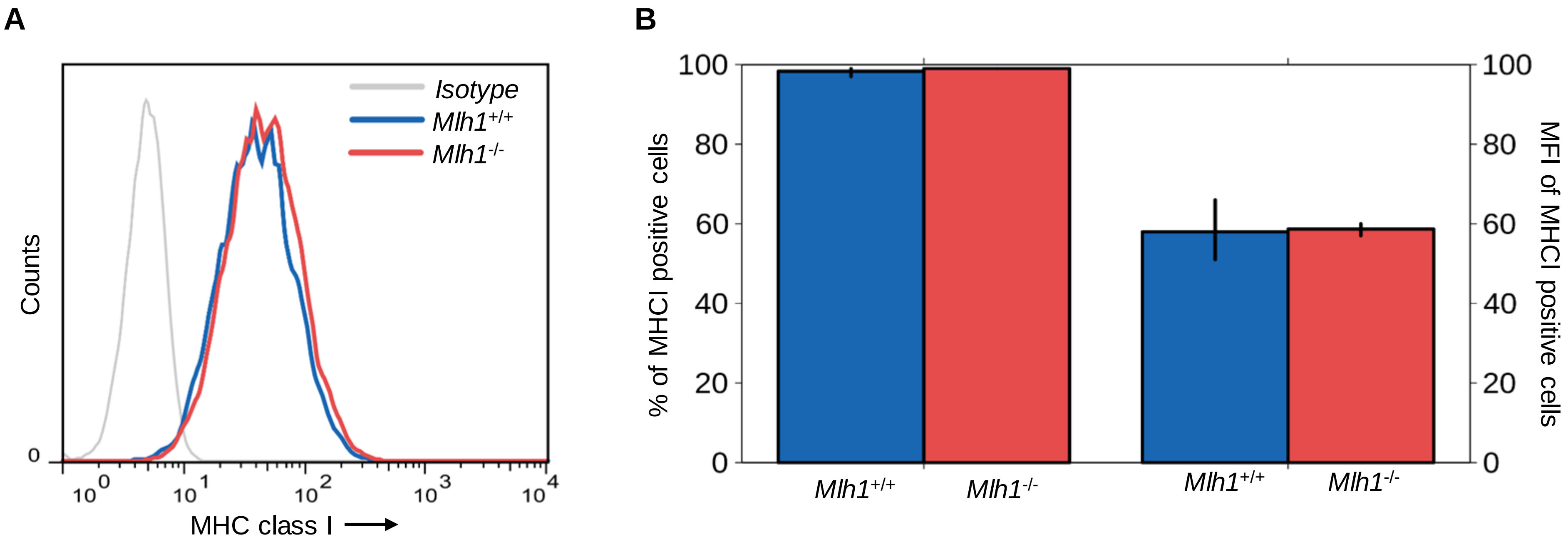

Fig S4: MHC class I levels in *Mlh1*$^{+/+}$ and *Mlh1*$^{-/-}$ cells. (A) MHC class I surface expression in CT26 *Mlh1*$^{+/+}$ and *Mlh1*$^{-/-}$ measured by FACS. The gray line corresponds to staining with an isotype control antibody. **(B)** The percentage of MHC class I positive cells and the mean of fluorescence intensity are shown for three biological replicates.

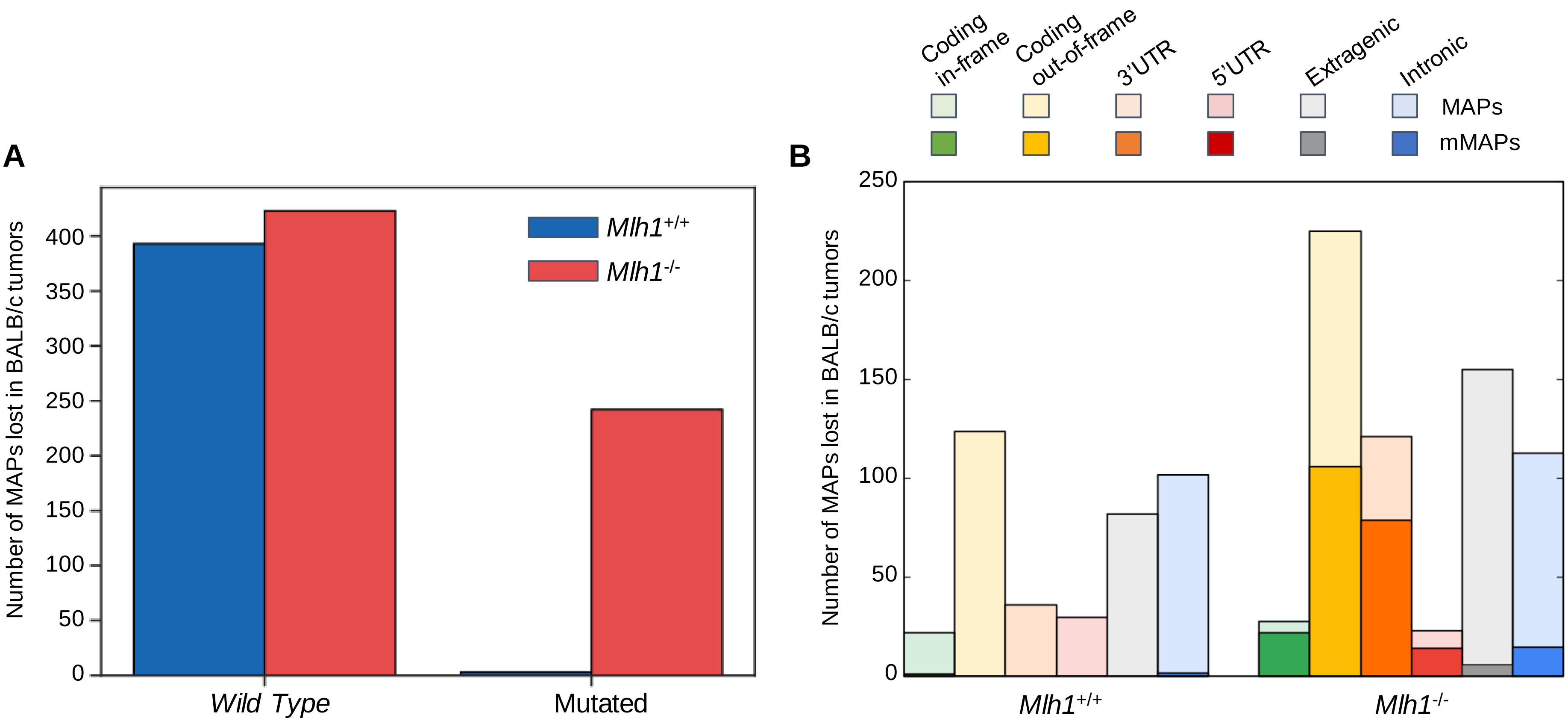

Fig S5: MMR-proficient and -deficient CT26 cells showed a high number of targeted non-canonical MAPs. (A) Total number of MAPs found in *Mlh1*$^{+/+}$ and *Mlh1*$^{-/-}$ tumors. The bar plot shows the aggregated number of MAPs in *Mlh1*$^{+/+}$ and *Mlh1*$^{-/-}$ samples. The bar groups are defined according to the mutational status of MAPs. **(B)** The number of MAPs annotated at genomic level in CT26 *Mlh1*$^{+/+}$ and *Mlh1*$^{-/-}$ samples are reported in light colors. mMAPs are highlighted in solid colors.

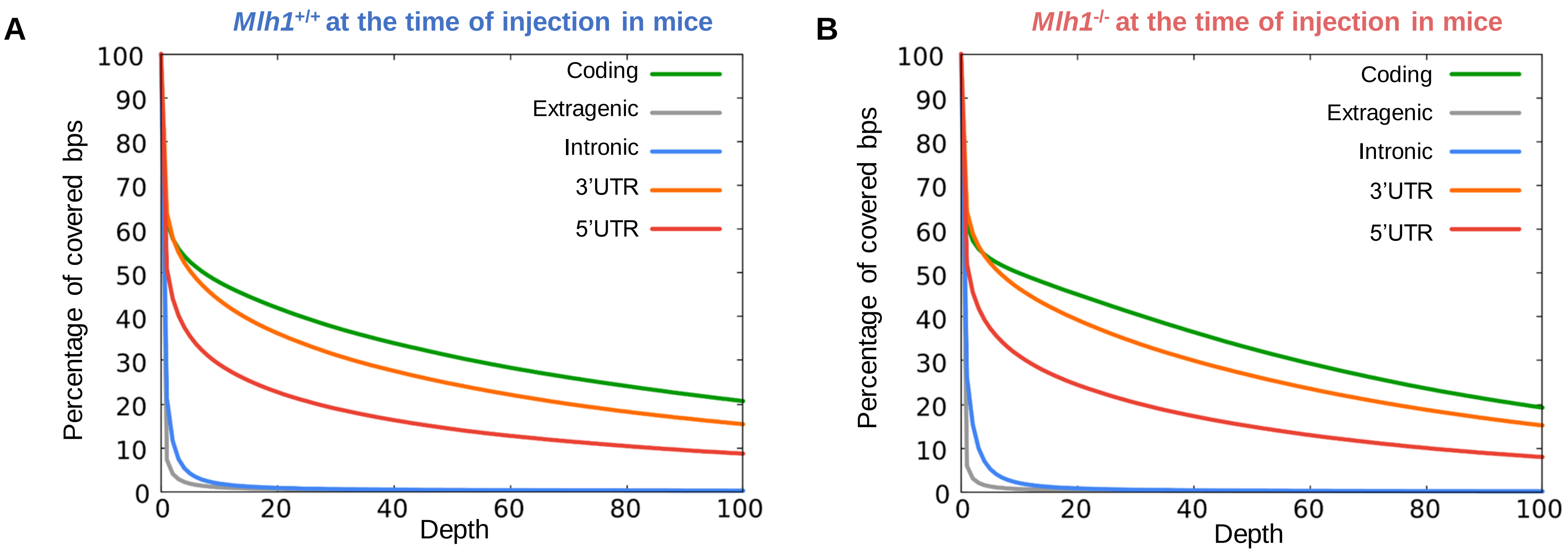

Fig S6: Coverage over depth analysis in all genomic regions of CT26 generated from RNA sequencing. The percentage of covered bases at single depth value resolution was calculated for each genomic region of *Mlh1*$^{+/+}$ **(A)** and *Mlh1*$^{-/-}$ RNAseq data **(B)**.

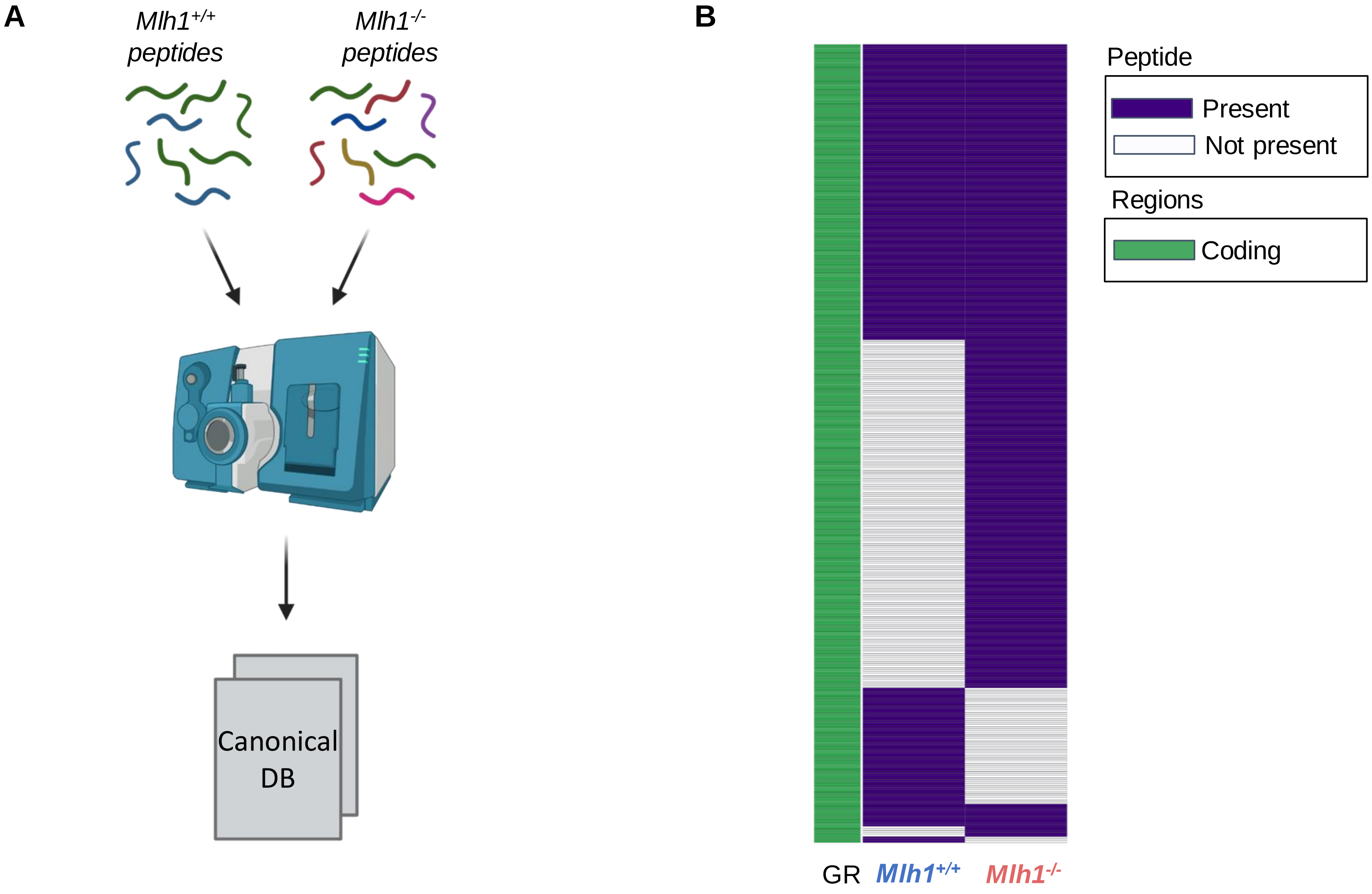

Fig S7: Identification of murine parental peptides. (A) Streamlined workflow of the immune-peptidomic pipeline in which MS spectra are matched to the UniProt mouse database. **(B)** Heatmap showing the peptide calls in CT26 *Mlh1*$^{+/+}$ and *Mlh1*$^{-/-}$ samples searching throughout the UniProt mouse database. The first heatmap column displays the genomic region (GR) annotation.

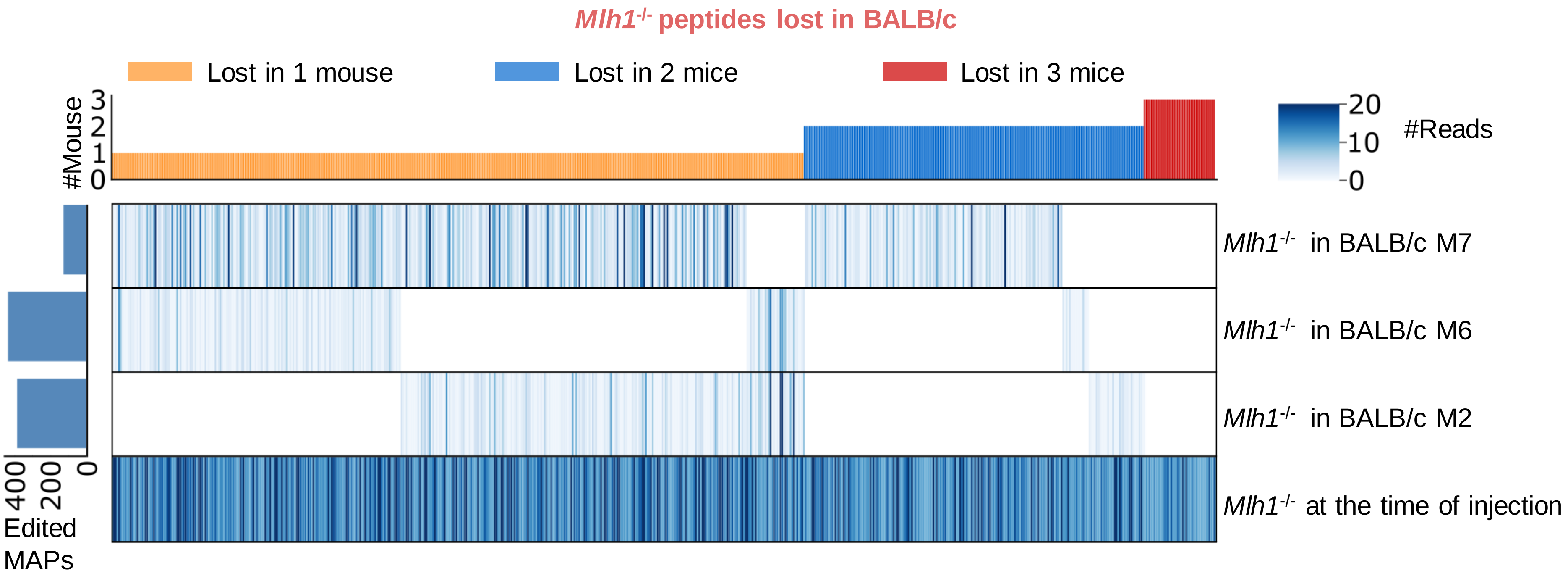

Fig S8: Expression variability of *Mlh1*$^{-/-}$ MAPs in immunocompetent mice. The number of RNA sequences supporting *Mlh1*$^{-/-}$ MAPs are showed in the heatmap: first three rows show transcript sequences found after tumor growth in BALB/c; last row shows transcript sequences present in *Mlh1*$^{-/-}$ cells at the time of injection. Bar plot on the heatmap top represents the number of mouse tumors in which MAP transcript sequences were absent. Bar plot on the left side shows in each tumor grown in BALB/c the number of MAPs that completely lost the allele expression.

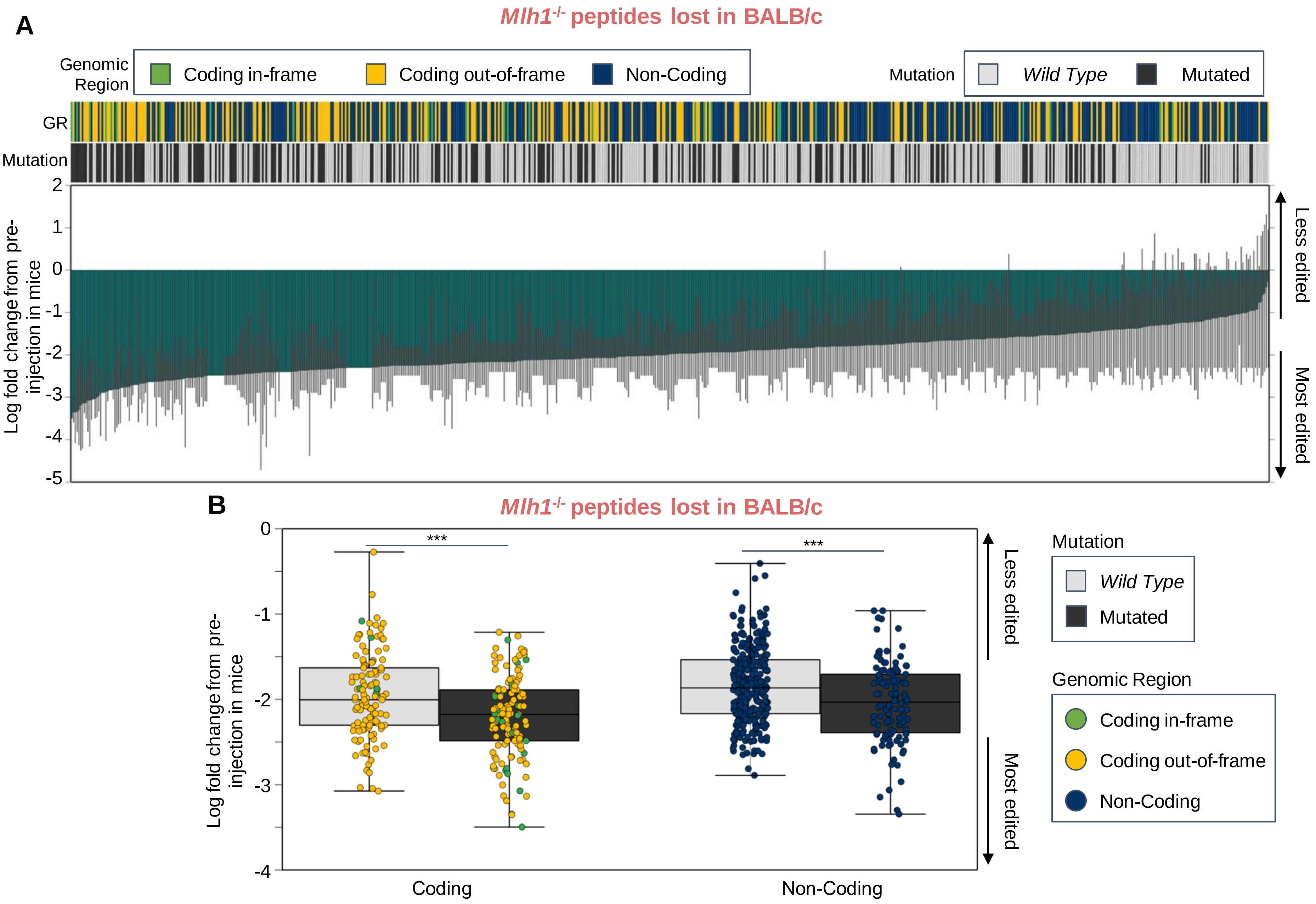

Fig S9: Immune editing of mutated MAPs in CT26 *Mlh1*$^{-/-}$ (A) Log fold change analysis performed between transcript values of CT26 *Mlh1*$^{-/-}$ peptides at the time of injection over those found after tumor excision from immunocompetent mice (dark green bars). The values are sorted from the lowest, i.e., the most targeted MAP, to the largest one, that is the least targeted. In gray, the standard deviation among the three mice measurement is reported. **(B)** Log fold changes from pre-injection values were grouped according to the peptide mutational status (Independent samples T-test: *** p-value < 0.0005).
